# Allotopic expression of ND6 restores vision in a mitochondrial disease model

**DOI:** 10.1172/jci.insight.209108

**Published:** 2026-09-08

**Authors:** Cheng Ai, Huiying Li, Jing Wu, Tianwei Zhou, Jing Wang, Shao-Hui Pan, Jun Yu, Douglas C. Wallace, Min-Xin Guan

**Affiliations:** 1 Center for Mitochondrial Biomedicine and Department of Ophthalmology, the Fourth Affiliated Hospital of School of Medicine, Zhejiang University, Yiwu, Zhejiang, China.; 2 Center for Genetic Medicine, International School of Medicine and International Institute of Medicine, and; 3 Institute of Genetics, Zhejiang University, Hangzhou, Zhejiang, China.; 4 Eye Research Center, Hangzhou Institute of Medicine, Chinese Academy of Sciences, Eye Hospital, Wenzhou Medical University, Hangzhou, China.; 5 Center for Mitochondrial and Epigenomic Medicine, Children’s Hospital of Philadelphia, Philadelphia, Pennsylvania, USA.

**Keywords:** Genetics, Ophthalmology, Gene therapy, Mitochondria, Retinopathy

## Abstract

Mutations in mitochondrial DNA (mtDNA) cause various mitochondrial diseases that are currently incurable. Allotopic expression of nucleus-recoded mitochondrial genes represents a promising therapeutic strategy, given its demonstrated capacity to restore mitochondrial function in human cell models harboring mtDNA mutations. However, the in vivo evaluation of allotopic gene therapy has been hindered by optimization challenges and the lack of appropriate animal models. Here, we overcome these limitations by utilizing an optimized AAV2-ND6 construct with codon optimization and mitochondrial targeting sequence in a mouse model bearing the homoplasmic ND6^P25L^ mutation, which recapitulates Leber hereditary optic neuropathy (LHON). High-dose administration of the AAV2-ND6 construct resulted in robust, sustained expression within the retina and optic nerve without apparent systemic toxicity. Strikingly, we compared the therapeutic efficacy in mutant mice at different ages and presymptomatic intervention with AAV2-ND6 effectively attenuated disease progression, mitigated retinal cellular deficiencies and optic nerve damage, and restored visual function in ND6^P25L^ mice. Mechanistically, allotopic ND6 expression markedly rescued the mitochondrial dysfunction, corrected dysregulated retinol metabolism and phototransduction pathways, and suppressed apoptotic processes in the mutant retina. Our study validates the safety and therapeutic potential of allotopic expression in vivo and provides critical mechanistic insights into its role in treating LHON and other mitochondrial diseases.

## Abstract 2

Allotopic expression of mitochondrial ND6 restored retinal and optic nerve function and improved vision in a mouse model of mitochondrial disease.

## Graphical abstract

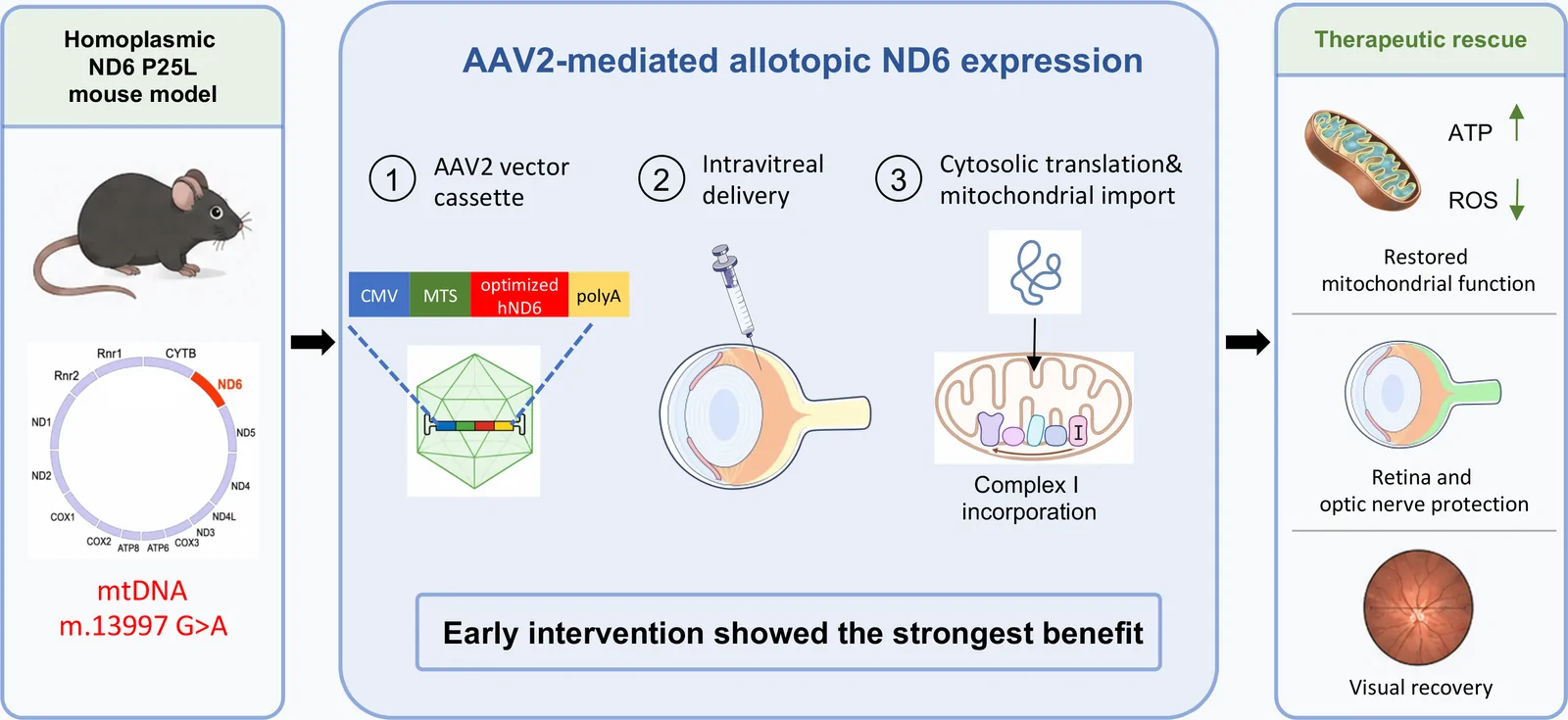

## Introduction

Mitochondrial diseases comprise a heterogeneous group of genetic disorders caused by mutations in genes encoded by either the nuclear or mitochondrial genome (1–3). The human mitochondrial genome (mtDNA) is a circular, double-stranded molecule of approximately 16.6 kb that encodes 13 polypeptides essential for the oxidative phosphorylation (OXPHOS) system, along with 2 rRNAs and 22 tRNAs required for mitochondrial translation (4). However, the vast majority of mitochondrial proteins (~1500) are encoded by nuclear genes, synthesized in the cytosol, and subsequently imported into mitochondria (5, 6). Unlike nuclear DNA, mtDNA exists in multiple copies per cell, ranging from hundreds to hundreds of thousands (7–9). Mutations in mtDNA are associated with a broad spectrum of clinical manifestations, including neuromuscular disorders, cardiomyopathy, diabetes, as well as hearing and visual impairment (10–12). These mutations can occur in either heteroplasmy (mixture of wild-type [WT] and mutated molecules) or homoplasmy (all mutated molecules). Heteroplasmic mtDNA mutations, such as the tRNA^Leu(UUR)^ 3243A>G mutation associated with mitochondrial encephalomyopathy, lactic acidosis, and stroke-like episodes (MELAS) and the tRNA^Lys^ 8344A>G mutation linked to myoclonic epilepsy with ragged red fibers (MERRF), typically affect multiple organs or tissues (13, 14). In contrast, homoplasmic mutations, including the 12S rRNA 1555A>G mutation associated with deafness and the ND4 11778G>A and ND6 14484T>C mutations associated with Leber hereditary optic neuropathy (LHON), often exhibit tissue-specific phenotypes (15, 16). Currently, there is no definitive cure for mitochondrial diseases; treatment strategies primarily focus on symptom management, metabolic support, and lifestyle modifications (17–19). Emerging gene-based therapies offer promising avenues for intervention. Gene-editing technologies such as CRISPR/Cas9, transcription activator-like effector nucleases (TALENs), DddA-derived cytosine base editors (DdCBEs), and TALE-linked deaminases (TALEDs) have been developed to selectively target mutant mtDNA, enabling the preferential repopulation of WT genomes (20–23). However, clinical translation of these approaches remains challenging due to the rarity and heterogeneity of mitochondrial diseases, complexity of therapeutic development, and the need for efficient and precise mitochondrial delivery systems to enhance efficacy while minimizing off-target effects (24).

Gene replacement therapy enables the delivery of functional gene copies using viral vectors such as adeno-associated viruses (AAVs) to restore protein activity. Among these approaches, allotopic expression of mitochondrial genes has emerged as a promising strategy for the treatment of mitochondrial diseases. In this approach, mitochondrial genes are recoded into the universal genetic code to allow accurate translation in the cytoplasm, followed by import of the synthesized polypeptides into mitochondria, thereby restoring mitochondrial function (25, 26). The efficiency of expression and precise mitochondrial localization of exogenous proteins depend on several key optimization strategies, including the refinement of 5′ mitochondrial targeting sequences (MTSs), 3′ untranslated regions (UTRs), and codon usages (27, 28). Notably, re-engineered ATP6 and ATP8 proteins have been successfully imported and incorporated into complex V, while ND6 has been shown to integrate into complex I and rescue mitochondrial dysfunction in ex vivo human cell models (29, 30). However, the in vivo efficacy and safety of allotopic expression remain insufficiently explored, largely due to the lack of appropriate animal models. Existing models, such as LHON rodent models generated by introducing exogenous nucleus-encoded mutant human ND4, typically retain endogenous WT mitochondrial genes (31–33). This genetic redundancy creates a substantial discrepancy from the genetic context observed in patients and limits translational relevance.

A mouse model harboring a homoplasmic LHON-associated ND6 13997G>A (P25L) mutation provides a robust platform for elucidating disease mechanisms and evaluating allotopic expression strategies in vivo (34, 35). LHON, the first described mitochondrial disease and the most common maternally inherited optic neuropathy, is characterized by acute or subacute central vision loss in young adults due to degeneration of retinal ganglion cells (RGCs) and their axons (36–39). Pathogenic mutations in mtDNA-encoded complex I subunits, particularly ND1, ND4, and ND6 (NADH:ubiquinone oxidoreductase), contribute to disease pathogenesis by impairing ATP production, increasing reactive oxygen species (ROS) generation, and triggering apoptosis (40–42). In this study, we investigated whether AAV2-mediated allotopic expression of ND6 could rescue visual dysfunction in ND6^P25L^ mice. We first conducted a dose-escalation study to evaluate the expression, localization, and systemic safety of an optimized ND6 construct in the mouse retina. Intravitreal injections were then administered to presymptomatic 2-month-old ND6^P25L^ and WT mice, followed by longitudinal in vivo assessment of retinal structure using optical coherence tomography (OCT). Structural and functional recovery were evaluated at 4 months after injection. We further examined the therapeutic efficacy of AAV2-ND6 when administered at the atrophic stage. Finally, we assessed the effects of allotopic ND6 expression on mitochondrial function, vision-related pathways, vitamin A metabolism, and apoptotic processes.

## Results

### Validation of AAV-mediated allotopic ND6 expression and localization in the mouse retina.

We optimized the allotopic expression of human ND6 (174 amino acids) by substituting 6 non-universal codons with universal codons and making 111 additional codon changes to enhance expression efficiency (Figure 1A and Supplemental Table 1; supplemental material available online with this article; https://doi.org/10.1172/jci.insight.209108DS1). To facilitate mitochondrial targeting, we appended a 25–amino acid MTS from cytochrome c oxidase subunit 8 (COX8) to the N-terminus of the ND6 open reading frame, resulting in the construct MTS-ND6. A FLAG-tagged version of this construct (MTS-ND6-FLAG) was also generated to allow for the validation of exogenous ND6 expression and localization in retinal cells. Both the FLAG-tagged and untagged ND6 constructs were packaged into AAV2 vectors, which are known for their efficacy in transducing retinal cells (43, 44).

We evaluated the expression of ND6 in the retina following intravitreal injection of AAV2-ND6 at 3 different doses: low (5 × 10^7^ vector genomes [vg]), medium (5 × 10^8^ vg), and high (5 × 10^9^ vg) into WT mice at 2 months of age. Especially, we administered the high dose to 2-month-old ND6^P25L^ mice. Three months after injection, the levels of exogenous ND6 were assessed using primer-specific PCR and Western blot analyses. As shown in Figure 1, B–E, robust and stable expression of exogenous ND6 was observed in both WT and ND6^P25L^ mouse retinas following high-dose injections of AAV2-ND6 or AAV2-ND6-FLAG. However, low- and medium-dose injections yielded lower expression levels in WT retinas. To determine whether exogenous ND6 was imported into mitochondria and assembled into complex I in vivo, we performed mitochondrial fractionation and blue native PAGE (BN-PAGE) immunoblotting using retinas from AAV2-ND6-FLAG–injected WT mice. ND6-FLAG was enriched in the mitochondrial fraction and detected within native complex I, supporting its successful mitochondrial import and incorporation (Figure 1, F and G).

Immunostaining of retinal cryosections from WT mice at 3 months after injection revealed that ND6-FLAG expression was detected in the RGC layer at the low dose, in the inner plexiform layer at the medium dose, and widespread across all retinal layers at the high dose, with the strongest signal in the peripapillary retina surrounding the optic nerve head (Figure 1H and Supplemental Figure 1, A and B). Dose-dependent expression patterns were also observed in the optic nerve of WT mice at 3 months after injection, consistent with the results of Western blot analysis (Figure 1I). However, viral genome analysis showed markedly lower vector genome levels in the optic nerve than in the retina, suggesting that much of the ND6 protein detected in the optic nerve may be synthesized in transduced retinal cells and subsequently transported along their axons (Supplemental Figure 1, C and D).

To assess the safety of varying AAV2-ND6 doses, we monitored body weight and examined retinal and major organ structures (heart, liver, spleen, kidney, intestine, and muscle) using hematoxylin and eosin (H&E) staining over multiple time points (Supplemental Figure 2A). No significant differences in body weight were observed, indicating that the AAV2-ND6 injections did not induce systemic toxicity (Supplemental Figure 2B). Furthermore, histological analysis of the retina and organs revealed no pathological alterations up to 6 months after injection, regardless of the dose (Supplemental Figure 2C and Supplemental Figure 3). These results indicated that AAV2-ND6 can express exogenous ND6 efficiently in the retina and did not have obvious side effects on retinal structure or other tissues with high-dose injection.

### Allotopic ND6 expression ameliorated retinal abnormalities.

Our previous study showed that the ND6^P25L^ mouse model develops optic neuropathy by 3 months of age (35). We investigated whether allotopic ND6 expression could prevent or restore retinal abnormalities associated with this condition. As outlined in the experimental timeline (Figure 2A), 2-month-old WT and ND6^P25L^ mice were intravitreally injected with either saline or a high dose of AAV2-ND6. We assessed retinal phenotypes at baseline (2 months) and subsequently at 3, 4, and 6 months using in vivo OCT. Subsequently, retinal and mitochondrial functions were evaluated.

The retinal phenotype of the ND6^P25L^ mice was further characterized. At 2 months, fundus examination revealed a normal appearance in ND6^P25L^ mice. However, by 3 months of age, ocular lesions began to appear, and these lesions progressively enlarged at 4 and 6 months, resembling the acute phase of LHON patients (Figure 2, B and C). OCT analysis further confirmed structural abnormalities in the retinal pigment epithelium and photoreceptor layers starting from 3 months (Figure 2D and Supplemental Figure 4). Quantitative analysis revealed that retinal thickness increased progressively from 3 to 6 months of age in the ND6^P25L^ mice (Figure 2E).

We first evaluated the therapeutic effect of the FLAG-tagged allotopic ND6. However, AAV2-ND6-FLAG did not improve the retinal phenotypes in the mutant retina and instead reduced RGC numbers in WT mice, possibly because the C-terminal FLAG interfered with the proper assembly or function of complex I (Supplemental Figure 5). We then intravitreally injected the untagged AAV2-ND6 or saline into 2-month-old ND6^P25L^ and WT mice. After 2 and 4 months of treatment, fundus and OCT analyses showed that retinal structures in AAV2-ND6–treated ND6^P25L^ mice were comparable to baseline (pretreatment) levels. In contrast, saline-treated ND6^P25L^ mice exhibited severe ocular lesions (Figure 2, B–D). Quantitative analysis further demonstrated that retinal thickness in the mutant retina was similar to those of WT retinas after both 2 and 4 months of AAV2-ND6 treatment (Figure 2E). Western blot and immunofluorescent staining analyses revealed retinal inflammation in both WT and ND6^P25L^ mice at 1 month after AAV2-ND6 injection. This inflammation was substantially attenuated at 4 months after injection (Supplemental Figure 6). Furthermore, we evaluated the therapeutic effect of AAV2-ND6 at the atrophy stage. Twelve-month-old WT and ND6^P25L^ mice were intravitreally injected with a high dose of AAV2-ND6 and assessed at 15 months of age (Supplemental Figure 7A). However, allotopic ND6 expression did not result in appreciable recovery of fundus lesion or retinal structure abnormalities in mutant retinas at 3 months after injection (Supplemental Figure 7B). These results demonstrated that presymptomatic intervention with allotopic ND6 expression effectively attenuated disease progression in the ND6^P25L^ mice.

### Rescued retinal cell deficiency.

We investigated whether allotopic ND6 expression rescued retinal cell–specific defects, including degeneration of RGCs, Müller cells, and rods, caused by the ND6^P25L^ mutation. ND6^P25L^ and WT mice, with or without AAV2-ND6 injection, were analyzed at 6 months of age using H&E staining and immunofluorescence assays. The H&E staining revealed that ND6^P25L^ mice exhibited structural abnormalities in the photoreceptor layers, along with a moderate reduction in the number of RGCs and photoreceptor nuclei (Figure 3, A–C). Notably, these retinal abnormalities were substantially ameliorated in the AAV2-ND6–treated group after 4 months of intervention (Figure 3, A–C). Immunofluorescent staining analysis further showed that allotopic ND6 expression markedly mitigated retinal cell degeneration in mutant mice, bringing them closer to levels observed in WT mice (Figure 3, D–H, and Supplemental Figure 8A). Specifically, the number of Brn3a-positive RGCs (a transcription factor essential for RGC survival) and the area of Vimentin-positive Müller glia process were restored to 134% and 187% of untreated mutants, respectively (Figure 3, D–G). Furthermore, ND6 overexpression corrected the abnormal distribution of rhodopsin in ND6^P25L^ mice (Figure 3H). No significant differences in retinal cell phenotypes were observed in WT mice, whether or not they received allotopic ND6 expression. Ultrastructural examination of the optic nerve using electron microscopy revealed axonal atrophy and demyelination in ND6^P25L^ mice. Figure 3I shows that the optic nerve axons of ND6^P25L^ mice exhibited severe demyelination. The average axonal density in ND6^P25L^ mice was reduced to 80.3% of that in WT mice (Figure 3J). Quantification analysis also revealed a significant increase in the number of extremely small axonal fibers (≤0.05 μm²) in ND6^P25L^ mice (Figure 3K and Supplemental Figure 8B). Importantly, allotopic ND6 expression substantially restored axonal structure, preserving axonal area and myelin integrity in the mutant mice (Figure 3, I–K). In contrast, allotopic ND6 expression administered at the atrophic stage did not restore degeneration of RGCs or rods in mutant retinas (Supplemental Figure 9). Together, these findings indicate that allotopic ND6 expression remarkably ameliorated retinal cellular deficiencies and optic nerve damage in ND6^P25L^ mutant mice when applied at a presymptomatic stage.

### Retinal function recovery.

We assessed whether allotopic ND6 expression resulted in visual function recovery after AAV2-ND6 injection for 4 months. Visual function was evaluated using full-field electroretinography (ffERG), visual evoked potentials (VEPs), and behavioral optomotor response (OMR). As shown in Figure 4, A and B, the scotopic (rod) b-wave amplitudes in ND6^P25L^ retinas with ND6 treatment were comparable to those in WT mice across all stimulus intensities (0.01, 3, and 10 cd·s/m²). In contrast, untreated mutant retinas exhibited a significant reduction in scotopic b-wave amplitudes. Figure 4, C and D show that allotopic ND6 expression also significantly enhanced the b-wave amplitudes of photopic (cone) responses in mutant mice, reaching up to 75.4% of the WT levels, compared to only 30.5% of WT levels in untreated mutants. Moreover, allotopic ND6 expression resulted in a 179% increase in oscillatory potentials, which reflect activity in the inner plexiform layer (45) in ND6^P25L^ mice compared with those in untreated mutant mice (Figure 4, E and F). To assess the integrity of the entire visual circuit, we performed VEP analysis. As shown in Figure 4, G and H, allotopic ND6 expression increased the N1-P1 amplitude in mutant mice to 241.6% of the untreated mutant levels, although still lower than that in WT mice. We also assessed the behavioral optomotor response by tracking the direction of rotation of the optokinetic (OKN) drum (46). As shown in Figure 4, I and J, WT mice initially exhibited an increase in OMRs followed by a decrease as spatial frequency increased, with the peak response at 0.2 Cyc/∞. In contrast, ND6^P25L^ mice showed no change in OMRs as frequency increased, maintaining the lowest levels. Importantly, allotopic ND6 expression led to a 29.8% increase in OMRs at 0.2 Cyc/∞ in the mutant mice, compared with untreated controls (Figure 4, I and J).

### Restoration of retinol metabolism and phototransduction pathways.

To systematically assess how allotopic ND6 expression restored retinal cellular deficits and visual function in ND6^P25L^ mice, we performed RNA-seq analysis on WT and mutant retinas with or without treatment at 4 months after injection. Principal component analysis (PCA) of the global data showed tight clustering of biological replicates across all 4 groups. Notably, ND6 treatment induced a marked shift in the transcriptomic profile of ND6^P25L^ retinas, while only mild changes were observed in WT retinas after treatment (Supplemental Figure 10A). Kyoto Encyclopedia of Genes and Genomes (KEGG) pathway analysis revealed a significant downregulation of retinol metabolism in the mutant retinas compared with WT mice, which is consistent with previously reported single-cell RNA-seq (scRNA-seq) findings (Figure 5A) (35). Remarkably, retinol metabolism was substantially upregulated in ND6-treated ND6^P25L^ retinas (Figure 5B). Both WT and ND6^P25L^ retinas injected with AAV2-ND6 also showed enrichment in pathways related to antiviral and xenobiotic responses, such as viral protein-cytokine interactions and cytochrome P450–mediated metabolism, indicating activation of a cellular defense response to AAV delivery (Figure 5B and Supplemental Figure 10B). Gene set enrichment analysis (GSEA) further indicated that allotopic ND6 expression restored the deficient retinol metabolism and exerted only mild effects on the downregulation of phototransduction pathways in ND6^P25L^ mutant retinas after 4 months of treatment (Figure 5, C and D). Indeed, retinol and its derivatives are critical for visual function, especially in regenerating the visual chromophore necessary for rhodopsin-mediated light reception, which triggers the phototransduction cascade to convert light into neural signals (47–49).

We further examined the effects of allotopic ND6 expression on retinol metabolism and phototransduction. RNA-seq data revealed upregulation of genes encoding retinoid dehydrogenases (*Rdh5*, *Rdh10*, and *Aldh1a2*) and visual cycle enzymes (*Rpe65* and *Lrat*), along with downregulation of the retinol degradation enzyme *Cyp26b1* in mutant retinas after allotopic ND6 expression (Figure 5E). Western blot analysis confirmed the upregulation of the retinol metabolism pathway, with increased levels of retinol metabolism–related components (RPE65, RDH5, RDH12, STRA6, RARB, and CRABP2) in AAV-injected ND6^P25L^ mice (Figure 5F and Supplemental Figure 10C). Furthermore, both RNA-seq and Western blot analyses showed the upregulation of phototransduction pathways, evidenced by increased levels of phototransduction components such as RHO, PRKCQ, PDE6B, GNGT1, and NMDAR1 (Figure 5, G and H, and Supplemental Figure 10D). These findings demonstrated that allotopic ND6 expression restored retinal cell deficiencies and visual function through activating retinol metabolism and phototransduction pathways.

### Rescued mitochondrial morphology and function.

To determine whether AAV-mediated allotopic ND6 expression can rescue mitochondrial dysfunction caused by the ND6 P25L allele, we measured the levels of ND6, ND1, and nucleus-encoded subunits of complex I (NDUFA13 and NDUFS2) using Western blot analysis. As shown in Figure 6, A and B, allotopic ND6 expression resulted in a 133.9% increase in ND6 levels in the mutant retinas compared with untreated mutants. In contrast, no significant differences were observed in the levels of ND1, NDUFA13, or NDUFS2 between mice with or without AAV2-ND6 injection. We then assessed whether allotopic ND6 expression restored mitochondrial ultrastructure by performing transmission electron microscopy. Mitochondria in the ganglion cell layer of mutant mice were enlarged and reduced in number, whereas mitochondria in the optic nerve appeared smaller and more numerous. As shown in Figure 6, C and D, ND6 treatment significantly ameliorated these mitochondrial abnormalities, including swelling, cristae malformations, and changes in size and number in both the ganglion cell layer of retina and optic nerve. We also evaluated mitochondrial function recovery in the retina using enzyme histochemical staining for NADH dehydrogenase (complex I), succinate dehydrogenase (SDH, complex II), and cytochrome *c* oxidase (COX, complex IV) in frozen retinal sections. As shown in Figure 6E, ND6 expression substantially increased complex I activity and modestly elevated SDH and COX staining in the mutant retina, indicating restored mitochondrial function. Furthermore, total cellular ATP levels in the mutant retinas increased to 142% of those in untreated mutant mice (Figure 6F).

Mitochondrial dysfunctions increase ROS production and therefore regulate the expression of antioxidant pathways (41, 50, 51). We measured the levels of antioxidant-related proteins, including catalase and superoxide dismutase (SOD1 and SOD2) in mouse retinas. As shown in Figure 6, G and H, the levels of SOD1, SOD2, and catalase in the mutant retinas were 148%, 62%, and 217% of those in WT retinas, respectively. Notably, ND6 treatment restored the levels of these antioxidant proteins in the mutant retinas to levels comparable to those in WT mice. We further examined the ratios of reduced glutathione to oxidized glutathione (GSH/GSSG) as an indicator of oxidative stress in the retina. As shown in Figure 6I, allotopic ND6 expression increased the GSH/GSSG ratio in the mutant retinas to 124% of that in untreated mutants, suggesting that ND6 expression effectively mitigated the overproduction of ROS.

### Reduced apoptosis.

Our previous study demonstrated that the ND6 mutation impaired cell viability and affected the apoptotic process in cybrids (30, 42). To further investigate the impact of the ND6^P25L^ mutation on apoptosis in vivo, we examined the expression of key genes involved in both intrinsic and extrinsic apoptotic pathways, antiapoptotic regulators, and the execution phase in RNA-seq data (Figure 7, A and B). ND6^P25L^ retinas at 6 months of age exhibited a marked upregulation of executioner caspases (*Casp3* and *Casp7*), intrinsic proapoptotic factors (*Bax*, *Bak1*, and *Casp9*), and a downregulation of the antiapoptotic gene *Bcl2*, compared with WT retinas. Notably, AAV-mediated ND6 expression restored the expression levels of these genes in ND6^P25L^ mice to levels comparable with those in WT mice. Furthermore, the ratio of *Bax* to *Bcl2*, a critical indicator of apoptosis (52), was significantly elevated in the mutant retina and substantially reduced after ND6 treatment, whereas no significant changes were observed in WT retinas following AAV injection (Figure 7C). We further assessed the impact of ND6 overexpression on apoptosis through Western blot analysis. As shown in Figure 7, D and E, 6-month-old ND6^P25L^ retinas displayed significantly increased levels of cytochrome *c*, BAD, BAX, caspase-3, and caspase-9, along with decreased levels of BCL-2, compared with WT retinas at the same age. Allotopic ND6 expression significantly reduced the levels of cytochrome *c*, BAD, BAX, and caspase 3, while increasing the levels of BCL-2 in the mutant retina, bringing them to levels comparable to those in WT retinas. We then evaluated the overall apoptosis levels using immunofluorescence analysis of retinal cryosections. As shown in Figure 7F, allotopic ND6 expression resulted in a significant reduction in cleaved caspase-3 levels in the ganglion cell layer and inner nuclear layer of ND6^P25L^ retinas, bringing the levels to those comparable to WT retinas. These data demonstrated that allotopic ND6 expression effectively reduced the overall apoptosis arising from the ND6 mutation.

## Discussion

Allotopic expression represents a promising therapeutic strategy for mitochondrial diseases, supported by its demonstrated ability to restore mitochondrial function in yeast and human cell models harboring mtDNA mutations (25, 26, 29, 30). However, its therapeutic efficacy depends on several critical optimization parameters, including the refinement of 5′ MTSs, 3′ UTRs, and nuclear codon usage (27, 28). A particularly important consideration is the divergence in polyadenylation mechanisms between nuclear and mitochondrial transcripts (53, 54). Notably, ND6 is unique among mtDNA-encoded genes in that its mature transcript lacks a poly(A) tail, potentially making it a more suitable candidate for allotopic expression (55, 56). Consistent with this, our previous work demonstrated that allotopic expression of ND6 effectively rescued mitochondrial dysfunction in cybrid cells carrying the LHON-associated m.14484T>C mutation (30). Using an optimized ND6 construct incorporating a COX8 MTS, we confirmed accurate signal peptide cleavage and restoration of complex I activity, as well as correction of apoptosis and mitophagy defects (30). Nevertheless, the lack of appropriate in vivo models, particularly those harboring homoplasmic LHON-associated mtDNA mutations, has limited evaluation of the safety and efficacy of this approach. To address this limitation, the present study extends our previous work from an in vitro cellular system to an in vivo homoplasmic mtDNA disease model, enabling the evaluation of retinal delivery, mitochondrial incorporation, long-term safety, stage-dependent therapeutic efficacy, and structural and functional outcomes in the visual system.

The homoplasmic ND6^P25L^ model provides several important advantages for studying LHON. Previous rodent models have been limited by “genetic redundancy,” as they retain endogenous WT mtDNA alongside introduced mutant genes, thereby confounding therapeutic evaluation (31–33). In contrast, the homoplasmic ND6^P25L^ model more closely recapitulates the genetic context of mtDNA-associated LHON and develops key disease-relevant phenotypes, including RGC loss, optic nerve axonal abnormalities, and marked visual dysfunction, thereby providing a robust platform for assessing disease progression and therapeutic response. Notably, the functional deficits, including marked reductions in ERG and VEP responses, were more pronounced than the relatively modest loss of RGC somata and photoreceptor nuclei. This discrepancy suggests that neuronal and axonal dysfunction may precede extensive neuronal loss and contribute substantially to visual impairment in this model. Nevertheless, the ND6^P25L^ model does not fully phenocopy human LHON. In particular, the outer retinal involvement and prominent ERG abnormalities observed in these mice extend beyond the classical clinical manifestations of LHON, which predominantly affect RGCs and the optic nerve (36–39). These differences may partly reflect inherent organizational and metabolic differences between the murine and human retina (57), while detailed characterization of retinal phenotypes in patients remains limited by the restricted accessibility of human retinal tissues. Despite these limitations, the homoplasmic ND6^P25L^ model provides a genetically relevant and experimentally valuable platform for investigating ND6-associated mitochondrial retinopathy and evaluating therapeutic interventions.

A key advance of this study is the development of an optimized AAV2-ND6 vector specifically engineered for efficient allotopic expression and mitochondrial delivery of human ND6 in vivo. In this construct, 6 non-universal codons and 111 additional codons were modified to enhance translational efficiency and stability. This construct achieved robust and sustained expression for up to 6 months following high-dose intravitreal injection. Importantly, exogenous ND6 was efficiently targeted to mitochondria and incorporated into complex I within the retina, with pronounced localization in the ganglion cell layer and optic nerve — tissues particularly vulnerable in LHON (36). These findings demonstrate that our optimized allotopic expression system enables durable, tissue-relevant gene delivery and successful incorporation into mitochondrial complex I in vivo. In this study, we further compared the therapeutic efficacy of AAV2-ND6 across different stages of intervention. While AAV-mediated allotopic expression has shown feasibility in clinical trials targeting ND4-associated LHON, therapeutic outcomes remain highly dependent on the timing of intervention (58–60). Notably, ND6^P25L^ mice developed acute fundus abnormalities by 3 months of age, defining a clear therapeutic window. Mice treated at 2 months of age exhibited significantly attenuated disease progression, as evidenced by reduced lesion areas and improved retinal architecture. Presymptomatic intervention with AAV2-ND6 also attenuated retinal and optic nerve degeneration, increasing the numbers of RGCs and photoreceptors, restoring the area of Müller glial processes, and preserving optic nerve myelination. Functional recovery was further supported by improvements in electrophysiological responses and optomotor behavior. In contrast, intervention at the atrophy stage resulted in minimal recovery of retinal deficits.

At the molecular level, this study provides in vivo evidence that allotopic expression rescues visual dysfunction accompanied by restoration of mitochondrial and cellular homeostasis. ND6 expression enhanced complex I activity, increased ATP production, and reduced ROS accumulation, consistent with our previous in vitro findings (30). Furthermore, mitochondrial morphology and distribution were normalized in both retinal and optic nerve tissues. Transcriptomic analyses further revealed that allotopic ND6 expression corrected dysregulated retinol metabolism and phototransduction pathways, which are essential for visual function. Retinol (vitamin A) metabolism is critical for photoreceptor maintenance and regeneration of the visual chromophore required for rhodopsin-dependent phototransduction (47–49), suggesting that restoration of these pathways may contribute to functional recovery. Furthermore, allotopic ND6 expression suppressed apoptosis in mutant retinas, as indicated by reduced levels of proapoptotic markers, including cytochrome *c*, BAX, BAD, and caspases, alongside increased expression of the antiapoptotic protein BCL-2. Interestingly, the gross retinal cell loss in mutant retina was relatively modest, suggesting that the apoptosis-related signaling may be activated before widespread neuronal death. In fact, in neurodegenerative disease, apoptotic signaling can also contribute to local degeneration of synapses and axons while neuronal somata remain temporarily preserved (61–63). Thus, the increased apoptotic signals observed in ND6^P25L^ retinas may contribute to the both synaptic and axonal abnormalities and the loss of a subset of retinal cells, while AAV2-ND6 treatment alleviated apoptosis and the associated retinal abnormalities. Although these molecular changes were consistent with improved retinal homeostasis after ND6 replacement, the present study does not establish whether alterations in retinol metabolism, phototransduction, or apoptotic signaling independently mediate the therapeutic effect. Collectively, these results indicate that allotopic ND6 expression reprograms cellular energy metabolism and prevents retinal cell degeneration induced by mtDNA mutations.

In summary, the evaluation of allotopic gene therapy in vivo has been constrained by challenges in optimization and the lack of physiologically relevant animal models. By combining an optimized AAV2-ND6 construct with a homoplasmic ND6^P25L^ mouse model, our study overcame these limitations and demonstrated that early intervention can effectively mitigated disease progression and restored retinal structure and function. These findings validated the safety and therapeutic potential of allotopic expression in vivo and provide critical mechanistic insights into its role in treating LHON and other mitochondrial diseases.

## Methods

### Sex as a biological variable.

Our study examined male mice because LHON is clinically more prevalent in males. Additionally, due to the reproductive challenges of mtDNA mutant mice, female mice were primarily used for breeding purposes.

### Mouse genetics.

All animal care protocols were approved by the Animal Care and Use Committee of Zhejiang University School of Medicine. C57BL/6J WT mice were originally purchased from Shanghai SLAC Laboratory Animal Co, Ltd. The mice harboring the mtDNA NADH dehydrogenase subunit 6 gene (ND6) 13997G>A P25L mutations were provided by Douglas C. Wallace (University of Pennsylvania, Philadelphia, Pennsylvania, USA), which were generated via the female embryonic stem cell fusion method (34, 64). The WT mice were maintained by brother-sister matings. The mtDNA ND6^P25L^ mice were maintained by crossing ND6^P25L^ female mice with WT males.

All experimental animals used were genotyped for both the *Crb1* rd8 allele and the *Nnt* allele to exclude C57BL/6N introgression or rd8 mutation–associated retinal degeneration (Supplemental Figure 11). PCR and Sanger sequencing of the *Crb1* locus did not detect the rd8 mutation in either the C57BL/6J WT mice or the ND6^P25L^ mice used in this study. PCR analysis did not detect the WT *Nnt* allele, which is characteristic of C57BL/6N-derived strains, in these mice (65). For genotyping of the ND6^P25L^ mutation, DNA samples isolated from tail biopsies were PCR amplified for ND6 gene and analyzed by direct Sanger sequencing. The primers are listed in Supplemental Table 2. All mice used for experiments were matched for sex and age.

### AAV production and injection.

The ND6 and ND6-FLAG constructs was packaged with serotype AAV2 and generated by PackGene. The titer of the produced AAV was 1 × 10^13^ vg/mL. For AAV delivery, WT mice received approximately 5 × 10^7^ vg, 5 × 10^8^ vg, or 5 × 10^9^ vg AAV per eye after dilution, and mutant mice received approximately 5 × 10^9^ vg AAV per eye after dilution via intravitreal injection. Mice were anesthetized with ketamine, and pupils were dilated by 1% tropicamide. The intravitreal injections were administered under an ophthalmic surgical microscope and a microliter syringe.

### Quantitative PCR.

Total RNA isolation and quantitative real-time reverse transcription PCR (qRT-PCR) were carried out as described previously (35). Briefly, the first-strand cDNA synthesis was carried out using a reverse transcription system kit according to the instructions of the manufacturer (AT311, Takara). qRT-PCR was performed using SYBR Green reagent (RK21220, ABclonal) on a CFX96 Real-Time PCR Detection System (Bio-Rad). The mRNA levels of hND6 were calculated after normalizing to β-actin. Primer sequences used for this analysis are presented in Supplemental Table 2.

For viral genome copy number analysis, total DNA of mouse retinas and optic nerves was isolated using a DNA isolation kit (DC112, Vazyme). The 2 retinas from each mouse were pooled as 1 retinal sample. For optic nerve analysis, 4 proximal or 4 distal optic nerve segments collected from 2 mice were pooled as 1 sample. qPCR was performed using SYBR Green reagent (RK21220, ABclonal) on a CFX96 Real-Time PCR Detection System (Bio-Rad). Relative viral genome copy numbers were calculated by normalization to the nuclear gene *Rpp30*. The primer sequences are provided in Supplemental Table 2.

### Western blot analysis.

Western blotting was performed using 20 μg of total cellular proteins prepared from mouse retinas, with 1 retina collected from each mouse, according to established protocols (66, 67). Proteins were separated in 10% Bis-Tris SDS–polyacrylamide gels and subsequently transferred onto polyvinylidene difluoride (PVDF) membranes. After incubation with primary antibodies and secondary antibodies, protein signals were detected using the ECL system (CWBIO). The quantification of density in each band was performed as detailed previously (66, 67). The antibodies used for this investigation are summarized in Supplemental Table 3.

### BN-PAGE analysis.

The incorporation of allotopic ND6 into complex I was assessed using mitochondria isolated from WT mouse eyecups at 3 months after AAV2-ND6-FLAG injection, as described previously (68). In brief, isolated mitochondria (400 μg), pooled from 30 eyecups of 15 mice per group, were solubilized on ice for 20 minutes in 0.5% DDM solution containing 50 mM NaCl, 50 mM imidazole, 2 mM 6-aminohexanoic acid, and 1 mM EDTA (pH 7.4). After removing insoluble material by centrifugation, samples were loaded onto 3%–11% acrylamide gradient gels and electrophoresed at 150 V in dark-blue cathode buffer for 1 hour and then 250 V in light-blue running buffer for 1.5 hours at 4°C. The native gels were transferred onto PVDF membranes at 30 V overnight for immunoblotting with antibodies listed in Supplemental Table 3.

### Fundus imaging and OCT.

A spectral domain OCT (SD-OCT) system (Micron IV, Phoenix Research Laboratories) was used to acquire fundus images and quantify retinal layer thickness. Mice were anesthetized with pentobarbital sodium, and mydriasis was induced by topical administration of 1% tropicamide (Bausch & Lomb). GenTeal Lubricant Eye Gel (Alcon) and Systane lubricant eye drops (Alcon) were applied to maintain corneal hydration throughout the procedure. Fundus visualization was achieved with an integrated imaging camera, and SD-OCT scans were acquired under real-time bright-field fundus guidance in accordance with the manufacturer’s protocol. Retinal layer thickness measurements were obtained using semiautomated analysis with Inviewer 3.0 software (Phoenix Research Laboratories).

### Immunofluorescence.

For whole flat-mount staining, 1 isolated whole retina from each mouse was fixed in 4% paraformaldehyde at room temperature for 2 hours and permeabilized with 0.2% Tween for 1 hour. Tissues were then blocked with FBS serum buffer (5% fetal bovine serum and 0.2% Triton X-100 in PBS) at room temperature for 1 hour and then incubated with primary antibodies at 4°C overnight, and then the secondary antibodies, as described elsewhere (69). Images were acquired using a Zeiss Axio Zoom.V16 and Leica DM4000B-M.

Immunostaining of retinal cross sections was performed as described previously (35). Eyecups were dissected and fixed in solution of 4% formaldehyde, 5% acetic acid, and 37.5% ethanol for 2 hours at room temperature and embedded in optimal cutting temperature compound (Thermo Fisher Scientific). Retinal transverse sections (12 μm) were permeabilized with 0.2% Triton X-100 in PBS for 1 hours and blocked with 3% fetal bovine serum for 1 hour at room temperature. Sections were then incubated with primary antibodies (Supplemental Table 3) at 4°C overnight, followed by the secondary antibodies. Fluorescence images were acquired using Leica DM4000B-M and Olympus Fluoview FV1000 microscopes. For immunofluorescence quantification, 1 eye from each mouse was used and 3 retinal sections were analyzed per eye to generate 1 biological replicate.

### H&E staining.

Mouse eyeballs were fixed with 4% paraformaldehyde at room temperature for 48 hours. Following fixation, tissues were dehydrated through graded alcohols, processed for paraffin infiltration, and embedded in paraffin blocks. Serial sections (3 μm) were cut using a rotary microtome (RM2235, Leica Microsystems) and subjected to H&E staining (BOSTER) for histological assessment of retinal architecture.

### Retinal ultrastructure and mitochondrial morphology.

Retinal ultrastructure was examined by transmission electron microscopy. Freshly dissected mouse retinas were fixed in 2.5% glutaraldehyde overnight, followed by postfixation with 1% OsO_4_ for 1 hour at room temperature. Samples were then stained and blocked with 2% uranyl acetate for 30 minutes, dehydrated through graded ethanol, and embedded in Epon 812 resin. Ultrathin sections (100 nm) were prepared using an UC7 ultramicrotome (Leica) and subsequently imaged with a TECNAI transmission electron microscope (Philips).

### Electroretinogram recordings and VEPs.

ERGs were acquired using a flash stimulation system integrated with an animal platform as detailed elsewhere (70). Briefly, mice were dark-adapted overnight and anesthetized with intraperitoneally injection of a ketamine/xylazine (80 mg/kg and 10 mg/kg, respectively). ERG signals were recorded using coiled silver wire electrodes placed in contact with the corneal surface. A subcutaneous gold needle electrode positioned between the shoulders served as both reference and ground. After completion of scotopic recordings, mice were light-adapted for 10 minutes prior to photopic ERGs and oscillatory potential acquisition. Scotopic ERGs were performed used flash intensities of 0.01, 3.0, and 10.0 cd·s/m², and photopic response and oscillatory potentials were recorded using 3.0 cd·m² flashes. For VEPs, the electrode was placed subcutaneously over the visual cortex, with recording performed at 1.6 Hz frequency over 150 repetitions.

### Quantitative optomotor response.

Optomotor responses were measured using a virtual reality system (qMOR) as previously described (71). Mice were placed on an elevated platform within a virtual rotating optokinetic drum. Visual acuity was determined by tracking head movements across a range of spatial frequencies (0.05–0.5 Cyc/deg), with tracking responses automatically quantified via qMOR software.

### RNA library construction.

Total RNA was extracted using TRIzol reagent kit (Invitrogen) according to the manufacturer’s protocol. RNA quality was assessed on an Agilent 2100 Bioanalyzer (Agilent Technologies) and checked using RNase-free agarose gel electrophoresis. After total RNA was extracted, mRNA was enriched by oligo(dT) beads and then fragmented into short fragments using fragmentation buffer and reversely transcribed into cDNA by using an NEBNext Ultra RNA Library Prep Kit for Illumina (NEB, 7530).

### RNA-seq analysis.

RNA-seq was performed using the Illumina HiSeq 4000 platform (Illumina). Sequencing reads were aligned to the mouse reference genome assembly GRCm38 using HISAT2 (version 2.2.1) with default parameters. Transcript assembly and gene-level quantification were conducted using StringTie (version 2.2.3) (72). Differential gene expression analysis was performed using DESeq2 (73), with genes exhibiting an absolute log_2_(fold change) greater than 0.5 and a *P* value less than 0.05 considered differentially expressed. Differentially expressed genes were subjected to enrichment analysis for KEGG pathways using the clusterProfiler package (74).

### Enzyme histochemistry.

Enzyme histochemical staining of the frozen sections from mouse retinas was performed as detailed elsewhere (35). Briefly, freshly dissected retina tissues were embedded in optimal cutting temperature compound (Tissue-Tek), frozen on dry ice, and sectioned at a thickness of 12 μm. For NADH dehydrogenase activity measurement, sections were incubated at 37°C for 60 minutes in Tris-HCl buffer (2 mM, pH 7.6) supplemented with 1.3 mM NADH and 3.06 mM NBT. For SDH staining, sections were incubated at 37°C for 25 minutes in 0.1 M phosphate buffer (pH 7.6) containing 5 mM EDTA, 1 mM potassium cyanide, 0.2 mM phenazine methosulfate, 50 mM succinic acid, and 1.5 mM nitro blue tetrazolium. For COX staining, sections were incubated at 37°C for 60 minutes in 5 mM phosphate buffer (pH 7.6) containing DAB (1 mg/mL), cytochrome *c* (1 mg/mL), and catalase (0.25 mg/mL).

### ATP measurement.

Total cellular ATP levels of mouse retinas were quantified using the Enhanced ATP Assay Kit (S0027, Beyotime) according to the manufacturer’s instructions with some modification, as described previously (75). Briefly, 1 retina of each mouse was processed to obtain total protein lysates, and ATP content was determined based on a luciferase-driven bioluminescence reaction. Luminescence signals were measured using a microplate reader and normalized to protein concentration.

### Detection of oxidative stress biomarkers.

The ratio of GSH/GSSG was evaluated using commercially available kit according to the manufacturer’s instructions (GSH and GSSG Assay Kit, S0053, Beyotime). One retina from each mouse was used.

### Statistics.

All data are expressed as mean ± SEM. Statistical analysis of the data was performed using GraphPad Prism 9. The statistical significance for multiple groups was assessed by 1-way ANOVA with Bonferroni’s post hoc test. Results are presented in dot plots, with dots denoting individual values. A *P* value of less than 0.05 was considered statistically significant. Further statistical details of experiments can be found in the Results and figure legends.

### Study approval.

All animal experiments were approved by the Institutional Animal Care and Use Committee, Zhejiang University School of Medicine.

### Data availability.

Raw RNA-seq data are available at the Big Sub, China National Center for Bioinformation (https://ngdc.cncb.ac.cn/gsub/), with the GSA accession number CRA040374. Values for all data points in graphs are reported in the Supporting Data Values file.

## Author contributions

MXG designed the experiments, monitored the project progression, and analyzed and interpreted data. CA, HL, and J Wu performed the mouse experiments. CA and J Wang designed and validated the ND6 construct. TZ and CA carried out RNA-seq data analysis. CA, J Wu, and TZ analyzed data. CA, HL, and SHP performed the vision function analysis. JY contributed to data analysis and interpretation and reviewed and edited the manuscript. DCW provided the mouse model. CA and MXG acquired funds. CA and HL prepared the initial draft of the manuscript. MXG produced the final version of the manuscript. All authors reviewed the manuscript.

## Conflict of interest

The authors have declared that no conflict of interest exists.

## Funding support

- National Natural Science Foundation of China (grants U25A20134 to MXG and 32500514 to CA).

## Supplementary Material

Supplemental data

Unedited blot and gel images

Supplemental table 1

Supporting data values

## Figures and Tables

**Figure 1:**
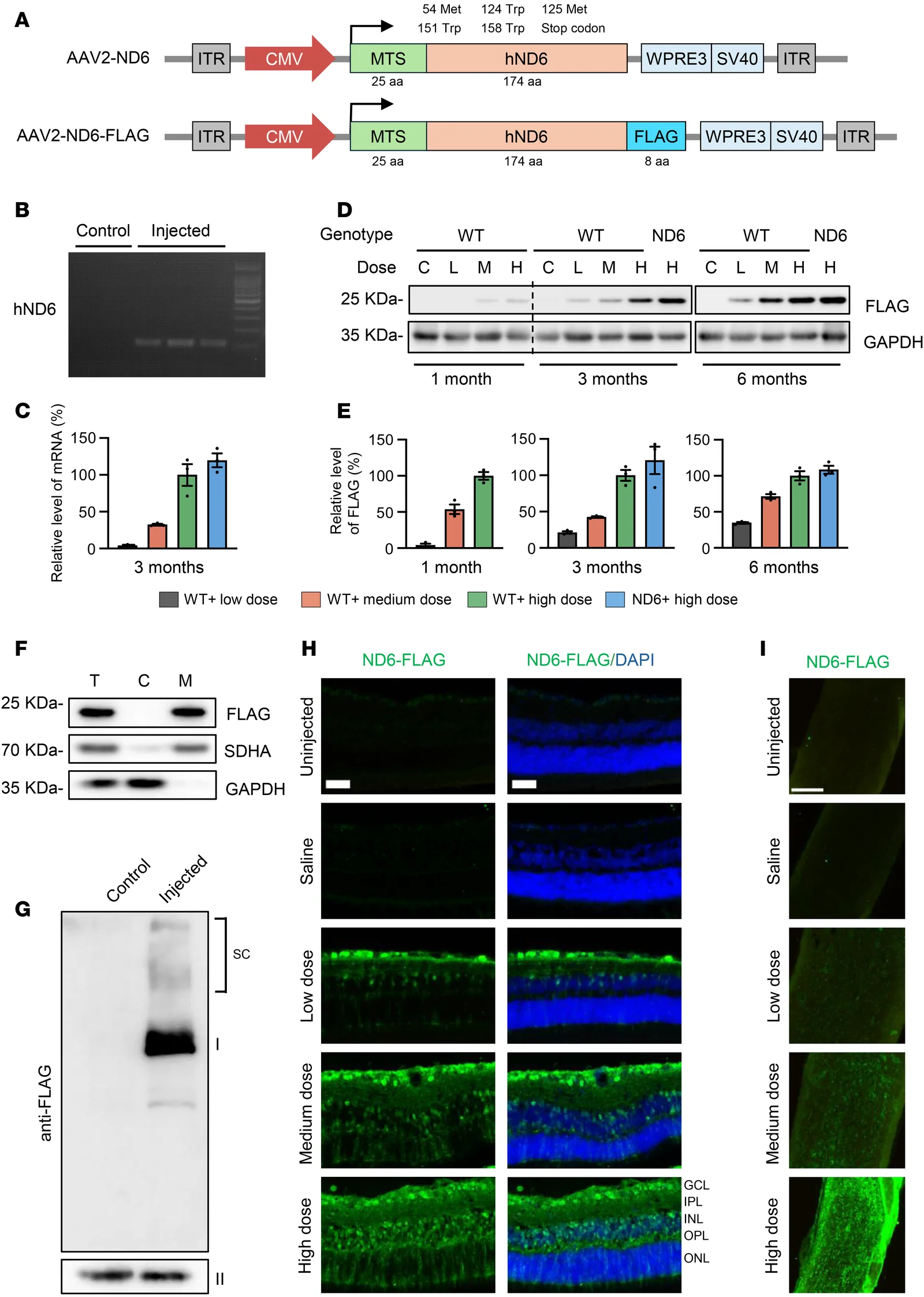
Expression and localization of modified ND6 in the retina. (**A**) Scheme for the structure of nuclear versions of human ND6. Six codons in mtDNA encoding ND6 were modified as universal codons and 111 additional codons were optimized as in Supplemental Table 1. The MTS of COX8 with 25 amino acids was added to the N-terminus of ND6. (**B**) Representative gel image showing the PCR product of allotopic ND6 in WT retina with or without AAV2-ND6 injection. (**C**) qPCR analyses of allotopic ND6 mRNA in WT and ND6^P25L^ retinas at 3 months after injection with different doses (*n* = 3 mice). (**D**) Western blot analysis of ND6-FLAG in WT and ND6^P25L^ retinas at 1 month, 3 months, and 6 months after injection. GAPDH was used as a loading control. (**E**) Quantification of ND6-FLAG in WT and ND6^P25L^ retinas at different time points (*n* = 3 mice). (**F**) Subcellular localization of ND6-FLAG in WT retinas assessed by mitochondrial and cytosolic fractionation and Western blotting with antibodies against FLAG, SDHA as a mitochondrial marker, and GAPDH as a cytosolic marker. T, total cell lysate; C, cytosol; M, mitochondria. (**G**) BN-PAGE immunoblot analysis of ND6-FLAG in WT retinas. Native mitochondrial protein were separated by BN-PAGE, transferred to membranes, and immunoblotted with antibodies against FLAG and SDHC (complex II). SC, respiratory supercomplexes. (**H**) Immunofluorescent staining of cryosection showing retinal sections at 3 months after injection, stained for FLAG (green) and with DAPI (blue). Scale bars: 50 μm. (**I**) Immunofluorescent staining of cryosection showing optic nerve section at 3 months after injection stained for FLAG. Scale bar: 100 μm. GCL, ganglion cell layer; IPL, inner plexiform layer; INL, inner nuclear layer; OPL, outer plexiform layer; ONL, outer nuclear layer. Data in **C** and **E** are shown as mean ± SEM.

**Figure 2:**
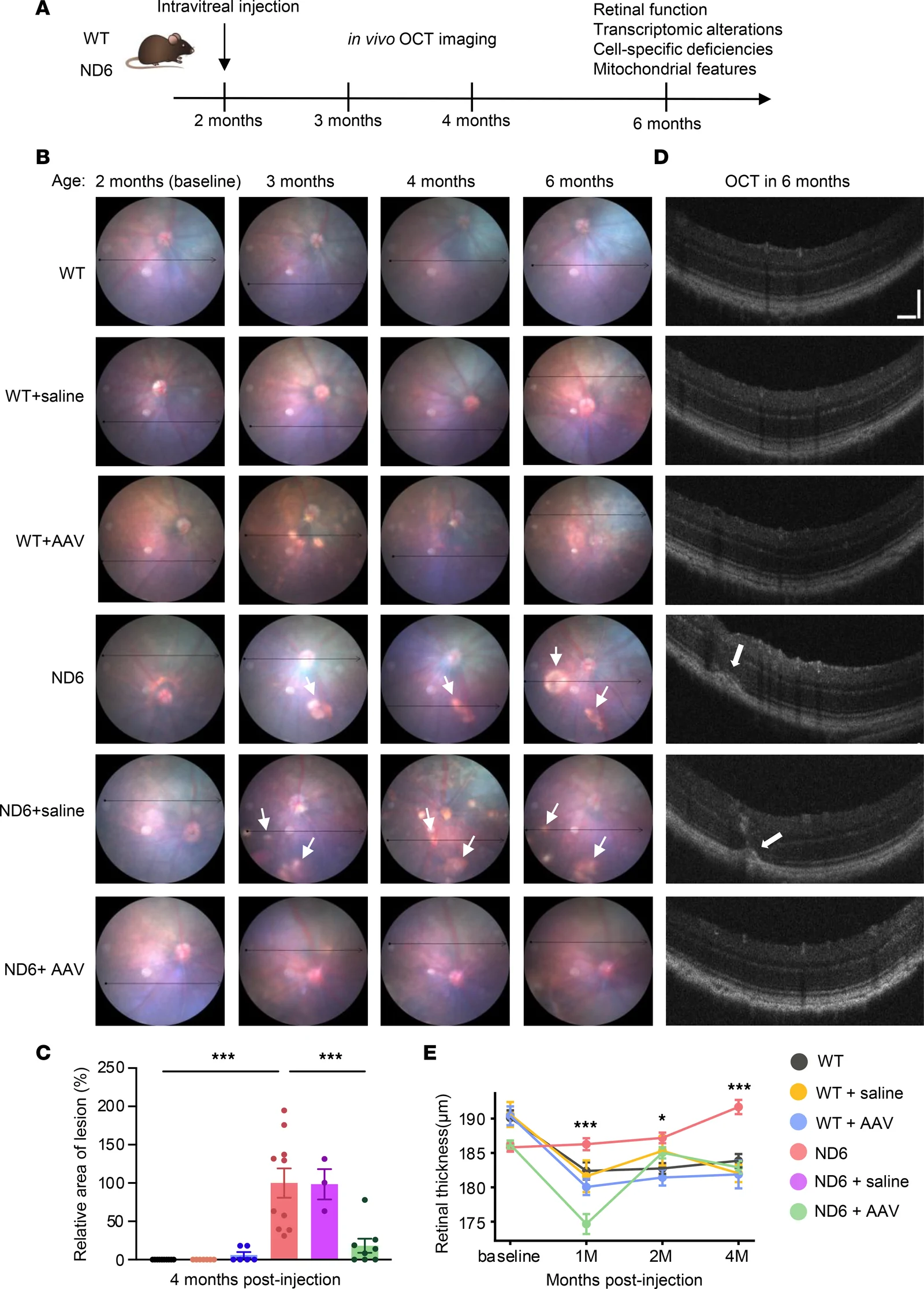
In vivo OCT analysis. (**A**) Schematic diagram of experimental procedures for the AAV-mediated allotopic ND6 intervention and pathological evaluation in WT and ND6^P25L^ retinas. The WT and ND6^P25L^ mice were divided into 6 groups: WT mice without injection (WT), WT mice with saline injection (WT+saline), WT mice with AAV2-ND6 injection (WT+AAV), ND6^P25L^ mice without injection (ND6), ND6^P25L^ mice with saline injection (ND6+saline), and ND6^P25L^ mice with AAV2-ND6 injection (ND6+AAV). (**B**) Image-guided OCT analysis was performed at 2 (baseline), 3, 4, and 6 months of age. The horizontal black arrows indicate the location of the OCT scans. White arrows indicate fundus lesions. (**C**) Quantification of relative area of lesion in WT (*n* = 10 mice), WT+saline (*n* = 7 mice), WT+AAV (*n* = 6 mice), ND6 (*n* = 10 mice), ND6+saline (*n* = 3 mice) and ND6+AAV (*n* = 8 mice) mice. (**D**) Representative OCT images of mouse retinas at 6 months of age. White arrows indicate abnormalities in the photoreceptor and the retinal pigment epithelium. (**E**) Quantification of retinal thickness at different time points after injection in various groups (*n* = 7–11 mice). *P* values in **E** compare ND6 and ND6+AAV mice. Data in **C** and **E** are shown as mean ± SEM. *P < 0.05; ***P < 0.001 by 1-way ANOVA with Bonferroni’s post hoc test.

**Figure 3:**
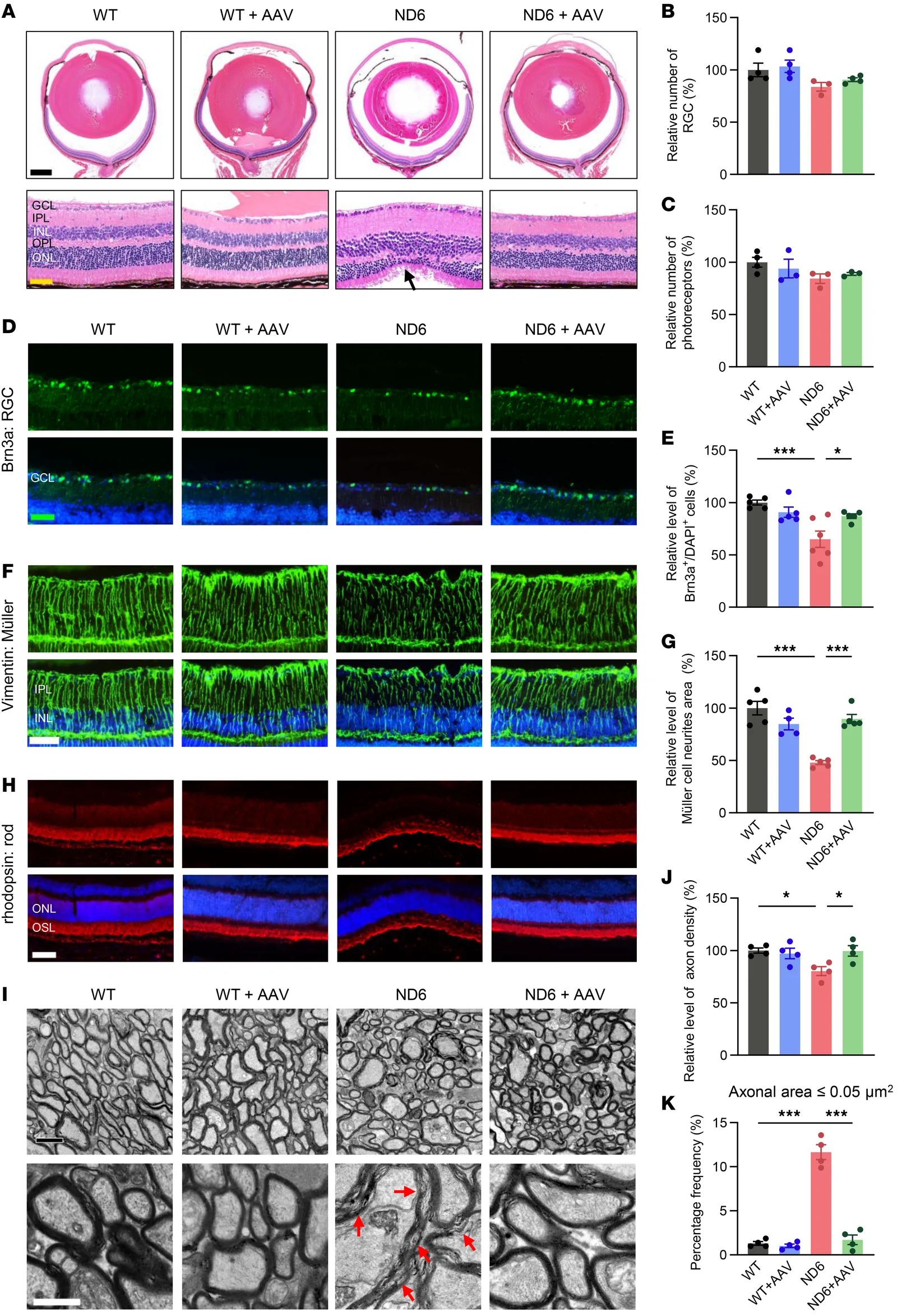
The recovery of retinal deficiency with allotopic ND6 expression. (**A**) Retinal section staining with H&E in various mice at 6 months of age. Lower panel showed the enlarged views to highlight layer alterations. The black arrow indicates an abnormality in the outer retina in ND6^P25L^ mice without treatment. Scale bars: 500 μm (upper panel) and 50 μm (lower panel). (**B** and **C**) The number of RGCs (**B**) and photoceptors (**C**) measured from H&E staining in various mice (*n* = 3–4 mice). (**D**) Immunofluorescent staining of cryosection showing retinal section stained for Brn3a (green) for RGCs and with DAPI (blue) for nuclei. Scale bar: 50 μm. (**E**) Quantification of ratios of Brn3a-positive RGCs (*n* = 5–6 mice). (**F**) Immunofluorescent staining of cryosection showing retinal section stained for Vimentin for Müller glia and with DAPI. Scale bar: 50 μm. (**G**) Quantification of relative area of Vimentin-positive Müller glial process area (*n* = 4–5 mice). (**H**) Immunofluorescent staining of cryosection showing retinal section stained with rhodopsin (red) and DAPI. Scale bar: 100 μm. (**I**) Ultrastructural analysis of RGC axons in optic nerve in various mice. The red arrows indicate the demyelination of axons in ND6^P25L^ mice. Scale bars: 2 μm (upper panel) and 1 μm (lower panel). (**J** and **K**) Quantification of axonal density (**J**) and percentage frequency of axon (area < 0.05 μm^2^) (**K**). *n* = 4 mice. Data in **B**, **C**, **E**, **G**, **J**, and **K** are shown as mean ± SEM. *P < 0.05; ***P < 0.001 by 1-way ANOVA with Bonferroni’s post hoc test. OSL, outer segment layer.

**Figure 4:**
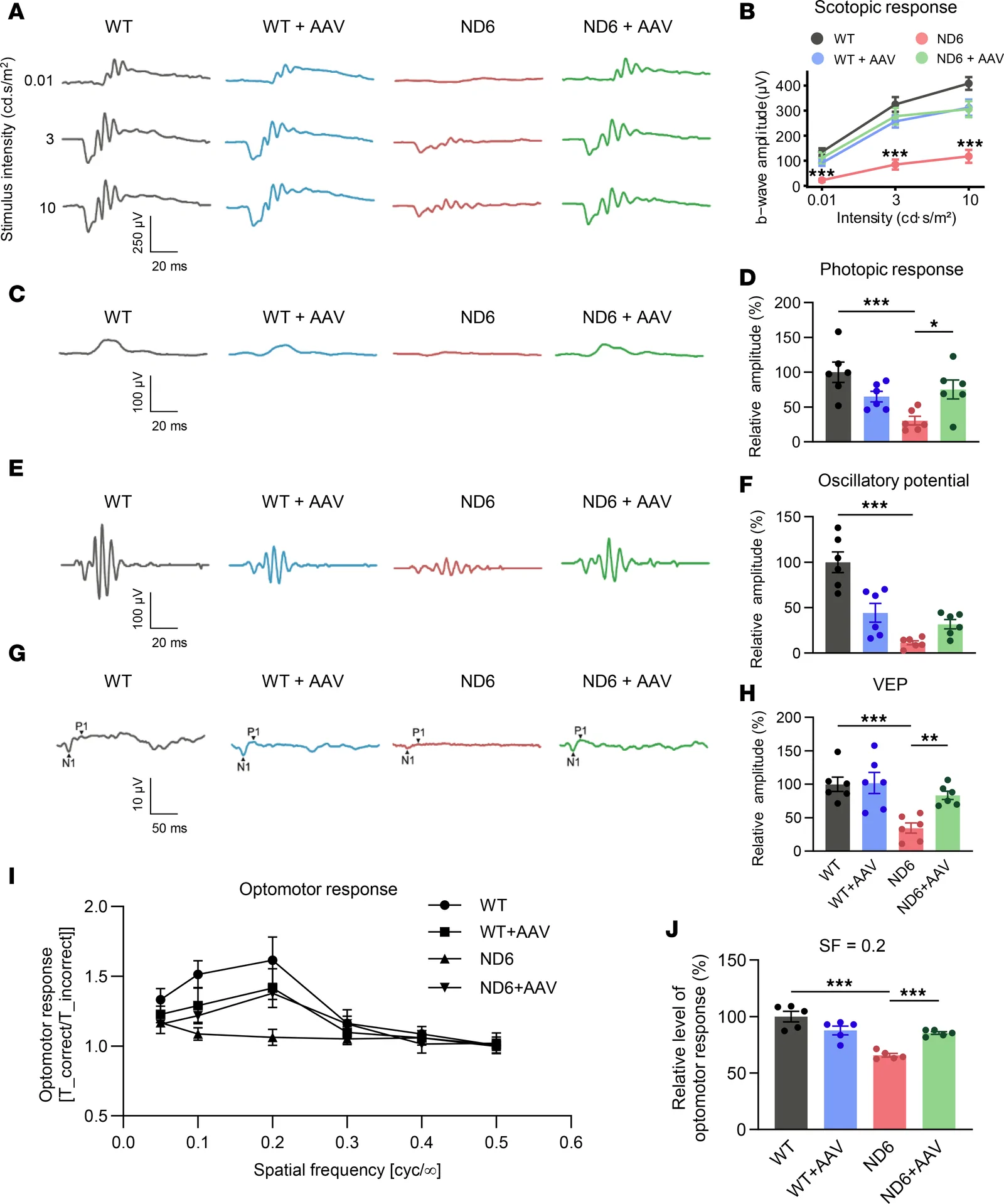
Visual function analysis. (**A** and **B**) Analysis of dark response in ffERG for WT, WT+AAV, ND6, and ND6+AAV mice (*n* = 6 mice). *P* values were calculated by 1-way ANOVA with Bonferroni’s post hoc test comparing ND6 and ND6+AAV mice. (**C** and **D**) Analysis of photopic response in ffERG for WT, WT+AAV, ND6, and ND6+AAV mice (*n* = 6 mice). (**E** and **F**) Analysis of oscillatory potential in ffERG for WT, WT+AAV, ND6, and ND6+AAV mice (*n* = 6 mice). (**G** and **H**) Analysis of visual evoked potentials (VEPs) for WT, WT+AAV, ND6, and ND6+AAV mice (*n* = 6 mice). Data are shown as mean ± SEM. (**I**) Analysis of optomotor response in different spatial frequency for WT, WT+AAV, ND6, and ND6+AAV mice (*n* = 5 mice). (**J**) Quantification of optomotor response in 0.2 Cyc/∞. SF, spatial frequency. Data in **B**, **D**, **F**, **H**, and **J** are shown as mean ± SEM. *P < 0.05; **P < 0.01; ***P < 0.001 by 1-way ANOVA with Bonferroni’s post hoc test.

**Figure 5:**
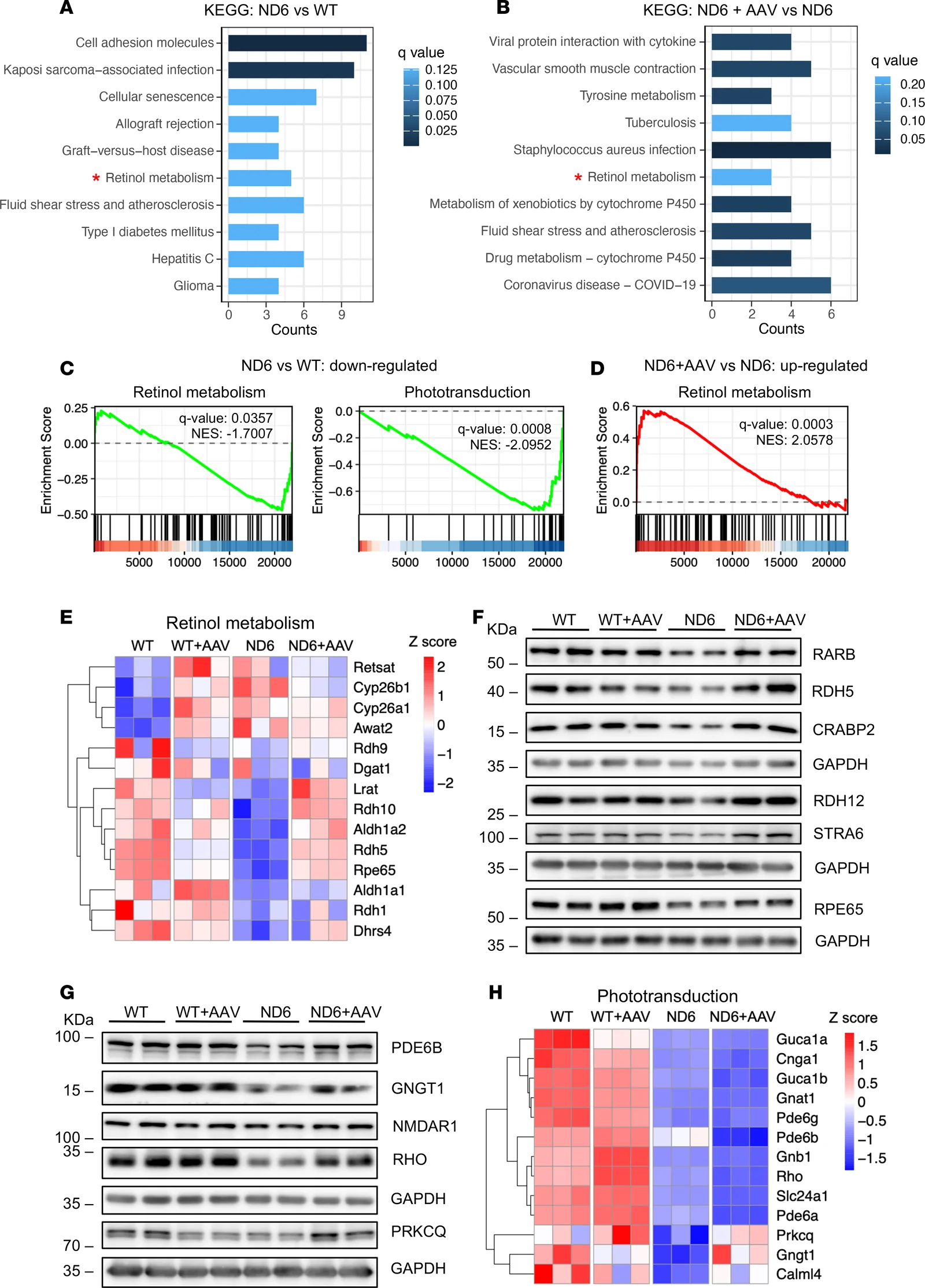
Transcriptome analysis of mouse retinas. (**A**) Bar plot showing KEGG pathway enrichment for downregulated genes in ND6 mice, as compared with WT mice. The *x* axis represented the number of differentially expressed genes and the color indicates *q* value. KEGG, Kyoto Encyclopedia of Genes and Genomes. (**B**) Bar plot showing KEGG pathway enrichment for upregulated genes in ND6+AAV mice, as compared with ND6 mice. The *x* axis represented the number of differentially expressed genes and the color indicates *q* value. (**A** and **B**) Red asterisks indicate the dysregulated retinol metabolism pathway. (**C**) Representative GSEA pathway that was significantly downregulated in ND6 mice, as compared with WT mice. GSEA, gene set enrichment analysis; NES, normalized enrichment score. (**D**) Representative GSEA pathways that were significantly upregulated in ND6+AAV mice, as compared with ND6 mice. (**E**) Heatmap displaying expression of genes involved in retinol metabolism by mean-centered log_2_ TPM in various mice. TPM, transcripts per kilobase million. (**F**) Western blot analysis of proteins involved in retinol metabolism (RARB, RDH5, RDH12, CRPBP2, STRA6, and RPE65). GAPDH was used as a loading control. (**G**) Western blot analysis of proteins involved in retinol metabolism (PDE6B, GNGT1, NMDAR1, RHO, and PRKCQ). GAPDH was used as a loading control. (**H**) Heatmap displaying expression of genes involved in phototransduction by mean-centered log_2_ TPM in various mice.

**Figure 6:**
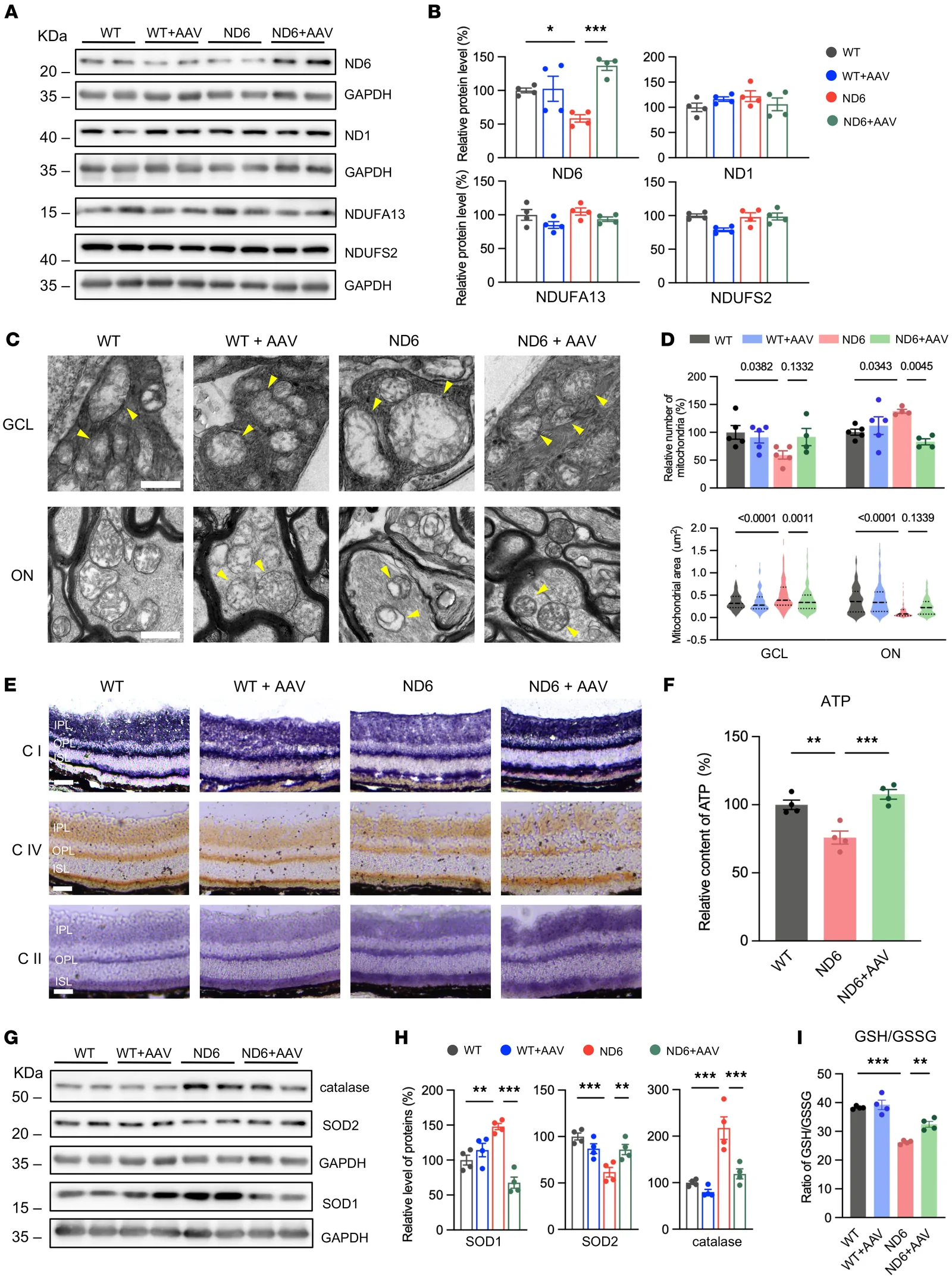
Mitochondrial morphology and function. (**A**) Western blot analysis of mitochondrial proteins with antibodies against ND6, ND1, NDUFA13, and NDUFS2, and GAPDH as a loading control. (**B**) Quantification of ND6, ND1, NDUFA13, and NDUFS2 in various mouse retinas. Representative of 4 independent experiments. (**C**) Representative transmission electron micrographs of mitochondria from the ganglion cell layer (GCL) of retinas and optic nerve (ON) in WT, WT+AAV, ND6, and ND6+AAV mice. Yellow arrows indicate mitochondria. Scale bar: 1 μm. (**D**) Quantification of mitochondrial size (*n* = 65–210 mitochondria) and relative mitochondrial number (*n* = 4–5 mice) in the GCL of mouse retina and ON. *P* values by 1-way ANOVA with Bonferroni’s post hoc test. (**E**) Assessment of mitochondrial function by enzyme histochemical staining for OXPHOS complexes I, II, and IV in the frozen sections of retinas in WT, WT+AAV, ND6, and ND6+AAV mice. Scale bar: 50 μm. (**F**) ATP levels among retinas of various mice (*n* = 4 mice) were measured using a luciferin/luciferase assay. (**G**) Western blot analysis of antioxidant proteins with antibodies of catalase, SOD1, and SOD2, and GAPDH as a loading control. (**H**) Quantification of catalase, SOD1, and SOD2 in WT, WT+AAV, ND6, and ND6+AAV retinas. Representative of 4 independent experiments. (**I**) The GSH content and GSH/GSSG ratio measured in various mouse retinas (*n* = 4 mice). Data in **B**, **D**, **F**, **H**, and **I** are shown as mean ± SEM. *P < 0.05; **P < 0.01; ***P < 0.001 by 1-way ANOVA with Bonferroni’s post hoc test.

**Figure 7:**
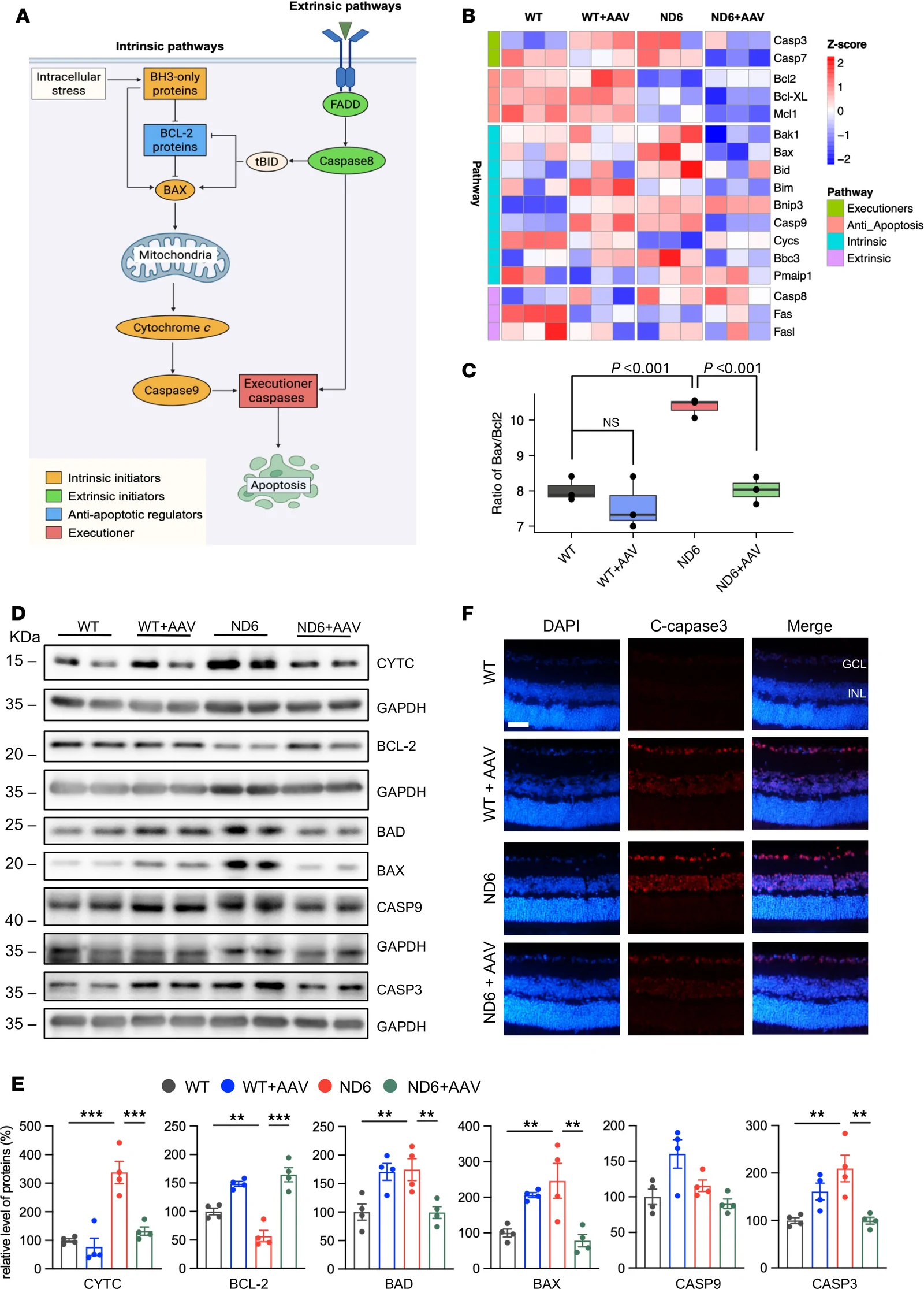
Apoptosis assays. (**A**) Schematic illustration of intrinsic and extrinsic apoptosis pathways. (**B**) Heatmap displaying expression of genes involved in apoptosis process by mean-centered log_2_ TPM in various mice. The specific apoptosis pathways are indicated by different colors. (**C**) Quantification of the TPM ratio of *Bax* to *Bcl2* in RNA-seq data (*n* = 3 mice). Boxes indicate the 25th–75th percentiles, center lines indicate medians, and whiskers indicate the minimum and maximum values. Black dots represent individual data points. (**D**) Western blot analysis of apoptosis-associated proteins. Twenty micrograms of total cellular proteins from various mouse retinas were electrophoresed, electroblotted, and hybridized with CYTC, BAX, BAD, BCL-2, and caspase-3 and -9 antibodies, with GAPDH as a loading control. (**E**) Quantification of 6 apoptosis-associated proteins. Representative of 4 independent experiments. Data are shown as mean ± SEM. (**F**) Fluorescence analysis of cleaved caspase-3 protein level in mouse retina at 4 month after injection. Cleaved caspase-3 is shown in red fluorescence, and DAPI-labeled nuclei are shown in blue fluorescence. **P < 0.01; ***P < 0.001 by 1-way ANOVA with Bonferroni’s post hoc test.

## Version 1 09/08/2026

In-Press Preview

## Version 2 10/08/2026

Electronic publication
