# Supplementary material for: Allotopic expression of ND6 restores vision in a mitochondrial disease model
Source: JCI Insight. 2026 Sep 8;11(19):e209108. doi: 10.1172/jci.insight.209108 (PMC13726423; doi:10.1172/jci.insight.209108)
Supplement: Supplementary file 1 [file jciinsight-11-209108-s262.pdf]

Supplementary data

Allotopic Expression of ND6 Restores Vision in a Mitochondrial Disease Model

Cheng Ai[1,2,3,#], Huiying Li[1,2,3,#], Jing Wu[1,2,3], Tianwei Zhou[1,2,3], Jing Wang[3], Shao-Hui Pan[4], Jun Yu[1,2,3], Douglas C. Wallace[5,*] , and Min-Xin Guan[1,2,3,*]

The supplemental data included the following information:

1. Supplemental Figure 1, 2, 3, 4, 5, 6, 7, 8, 9, 10, and 11
2. Supplemental Table 2 and 3

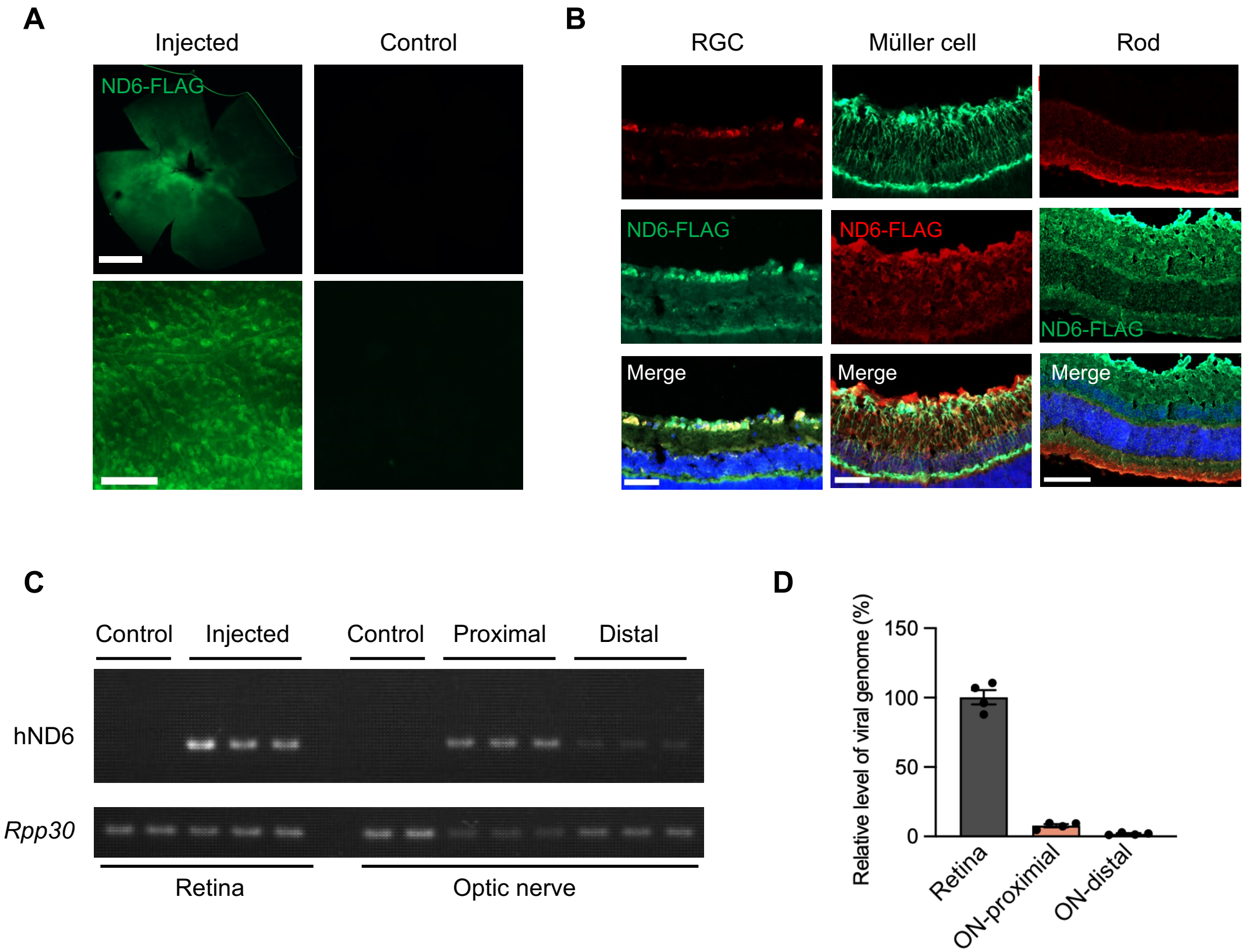

Supplemental Figure 1 (related to Figure 1): Validation of AAV transduction efficiency and localization. (A) Retinal whole-mount immunofluorescence staining with anti-FLAG antibody at 3 months after intravitreal injection of AAV2-ND6-FLAG. An uninjected retina was used as the control. Scale bars: 1 mm (upper panel); 100 µm (lower panel). **(B)** Co-immunofluorescence staining of retinal cryosections from WT mice at 3 months after AAV2-ND6-FLAG injection. Sections were stained for ND6-FLAG together with markers of RGCs (Brn3a), Müller cells (vimentin), or rods (rhodopsin). Nuclei were counterstained with DAPI. Scale bars: 50 µm (left and middle panels); 100 µm (right panel). **(C)** Representative PCR analysis of AAV2-ND6 vector genomes in uninjected and injected retinas and in control, proximal, and distal optic nerve segments. *Rpp30 was used* as the genomic DNA control. **(D)** qPCR quantification of AAV2-ND6 vector genomes in injected retinas and proximal and distal optic nerve segments at 3 months post-injection. Vector genome levels were normalized to *Rpp30.* Data are shown as mean ± SEM. n = 4 mice.

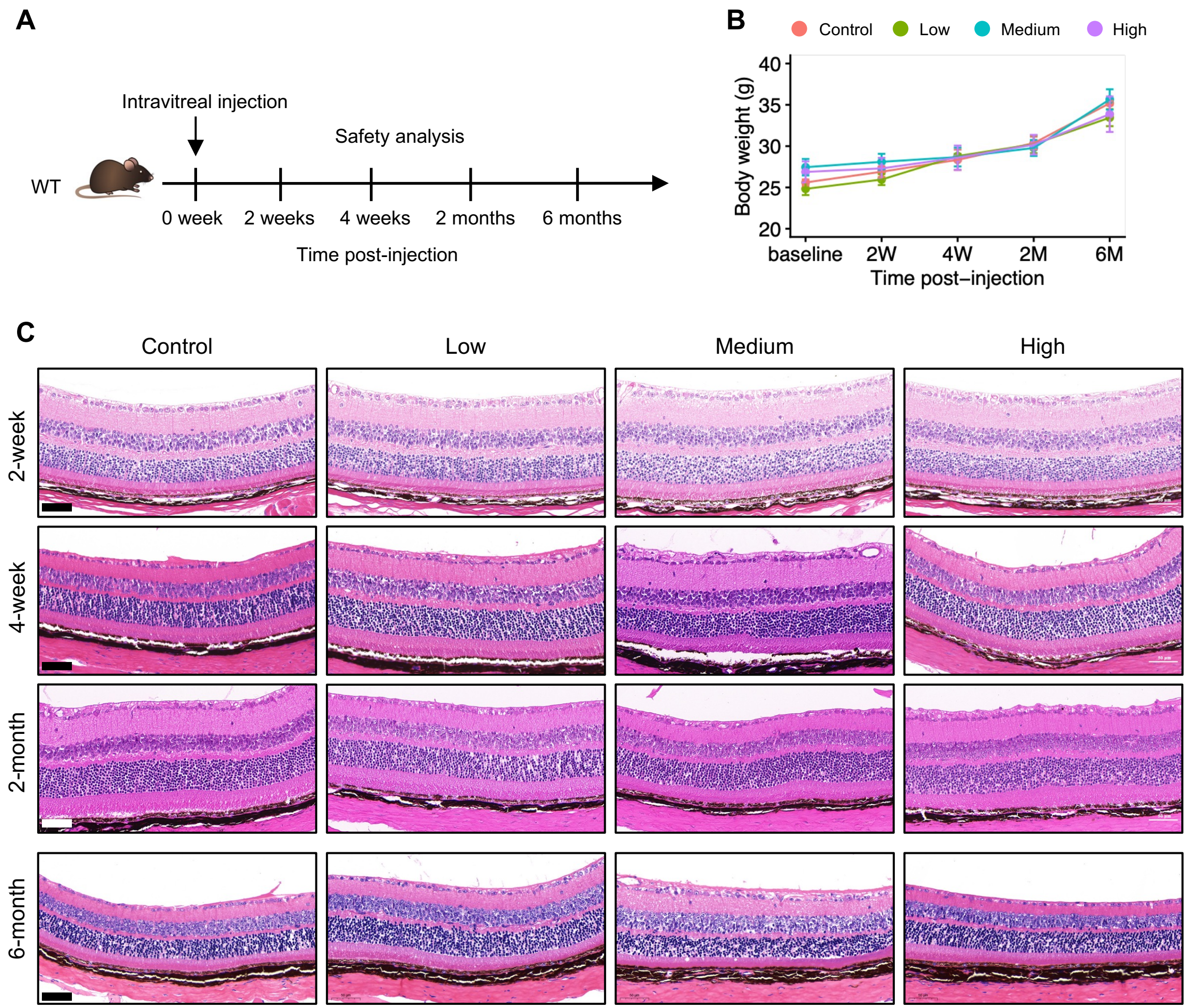

Supplemental Figure 2 (related to Figure 1): The safety assessment of AAV2-ND6 in WT mice. (A) Scheme of AAV2-ND6 treatment and safety analysis. Two-month-old WT mice received intravitreal injections of various doses of AAV2-ND6 (Low dose: 5×10^7 vg/eye; Medium dose: 5×10^8 vg/eye; High dose: 5×10^9 vg/eye). Safety assessments were performed at 2 weeks, 4 weeks, 2 months, 6 months post-injection. **(B)** Weight changes of mice in different groups (n = 4–5 mice). **(C)** Representative retinal sections staining with H&E of mice in different groups at different time points. Scale bar: 50 μm.

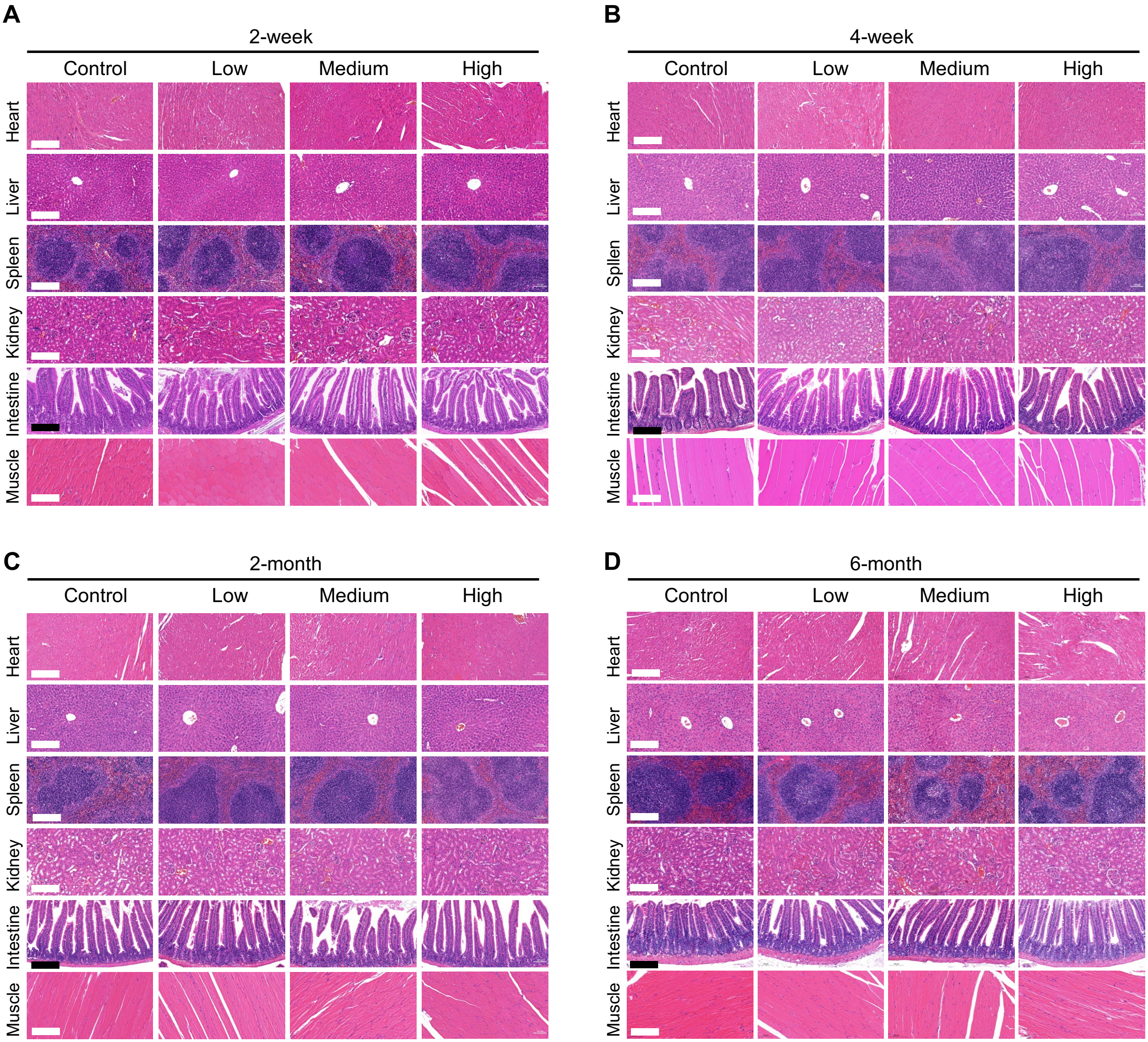

Supplemental Figure 3 (related to Figure 1): The safety assessment of AAV2-ND6 in major organs of WT mice. Organ index of WT mice injected with different doses of AAV2-ND6 at 2 weeks **(A)**, 4 weeks **(B)**, 2 months **(C)**, 6 months **(D)** post-injection. Scale bar: 200 μm.

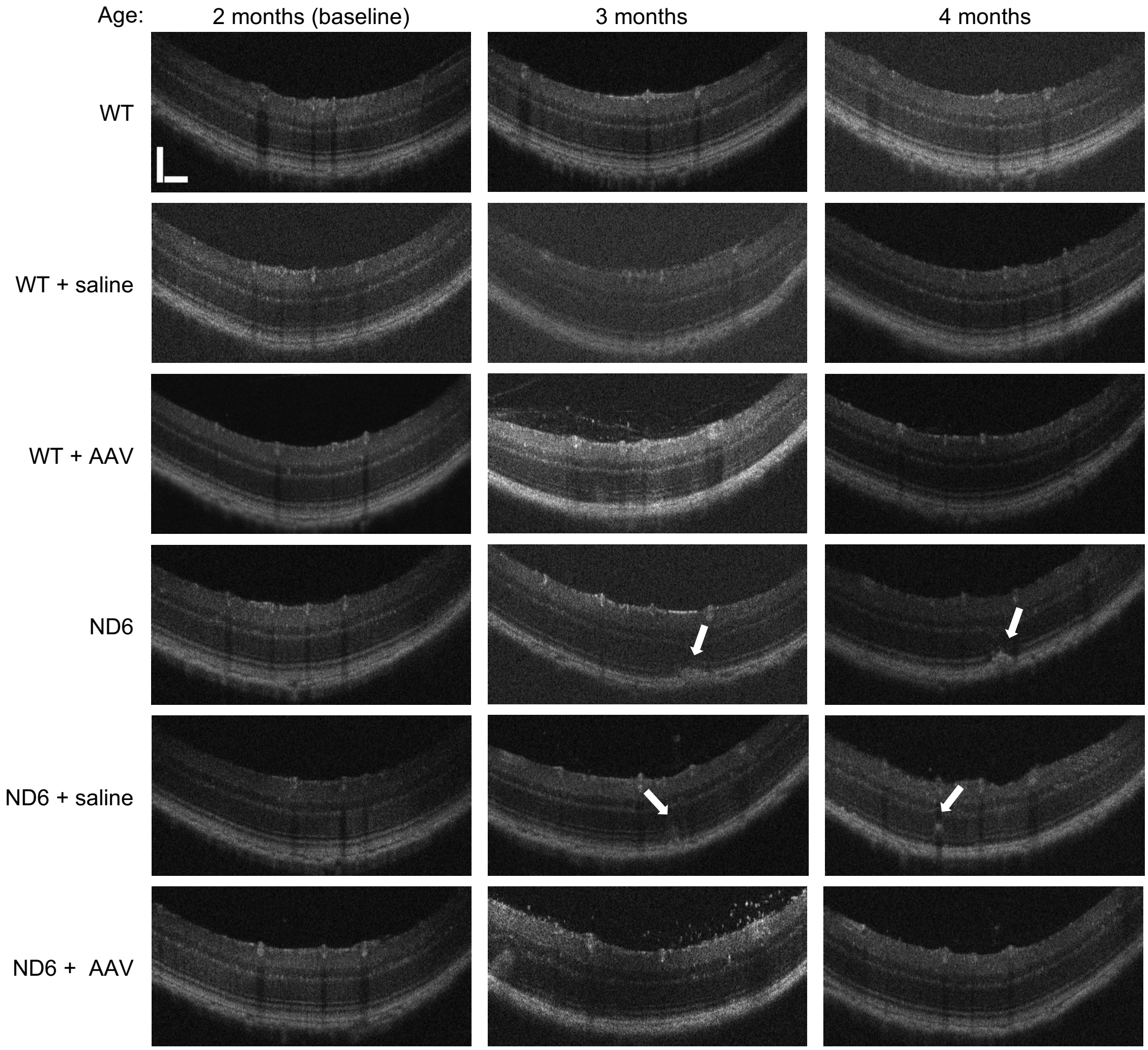

Supplemental Figure 4 (related to Figure 2). Representative OCT images of mice retina in at 2 months (baseline), 3 months and 4 months of age. The white arrow indicated abnormalities in the photoreceptor and RPE. Scale bar: 100 μm.

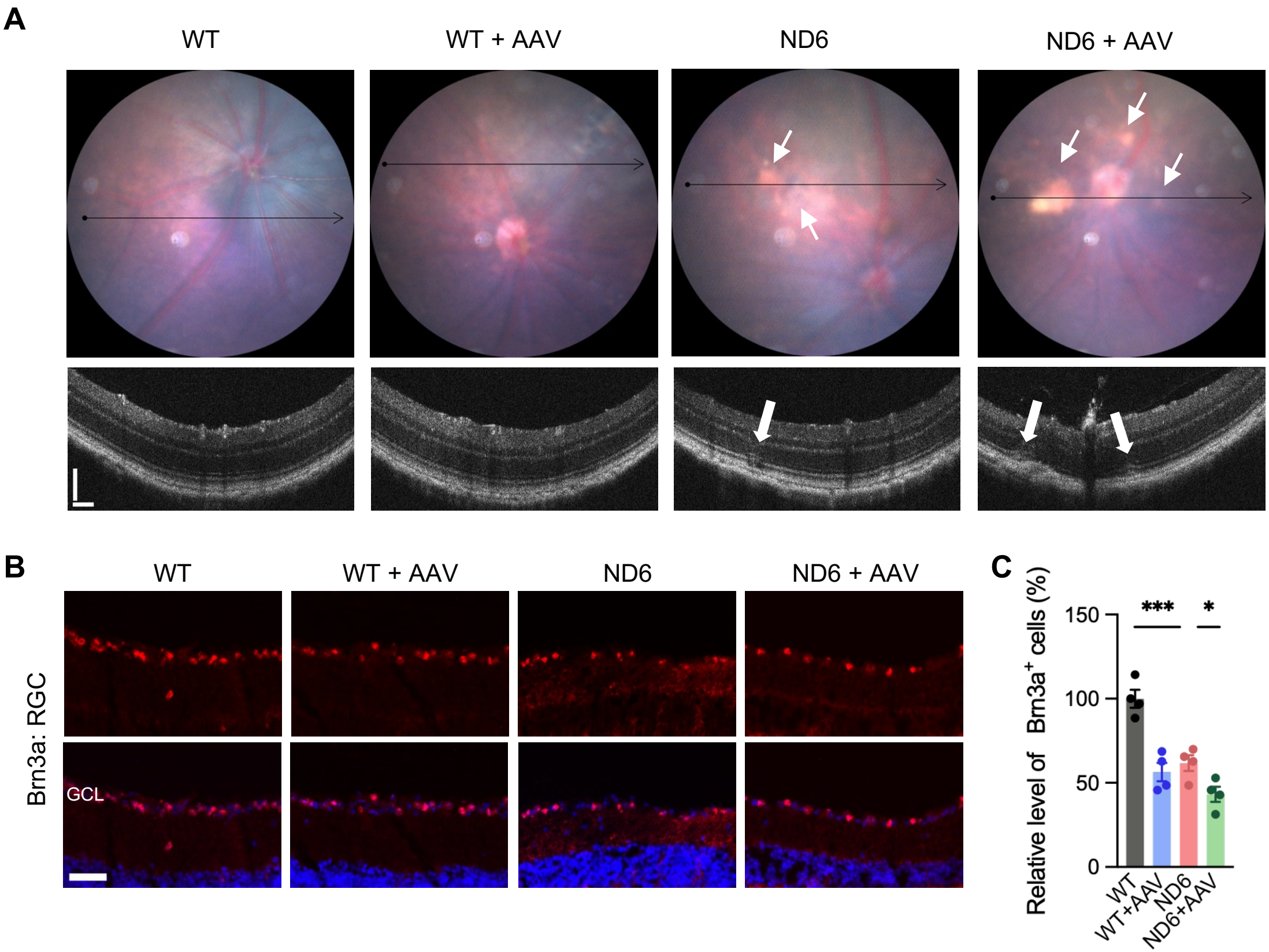

Supplemental Figure 5 (related to Figure 2). Therapeutic evaluation of AAV2-ND6-FLAG. Two-month-old WT and $ND6^{P25L}$ mice were intravitreally injected with a high dose of AAV2-ND6-FLAG. Retinal phenotypes were subsequently assessed at 5 months of age, 3 months post-intervention. **(A)** Representative OCT images of mice retina at 5 months of age. The white arrow indicated abnormalities in the photoreceptor and RPE. Scale bar: 100 µm. **(B)** Immunofluorescence staining of cryo-section showing retinal section stained with Brn3a (red) for RGC and DAPI (blue). Scale bar: 50 µm. **(C)** Quantification of Brn3a positive RGC levels (n = 4 mice). Data are shown as mean ± SEM. *P < 0.05, ***P < 0.001 by one-way ANOVA followed by Bonferroni post hoc test.

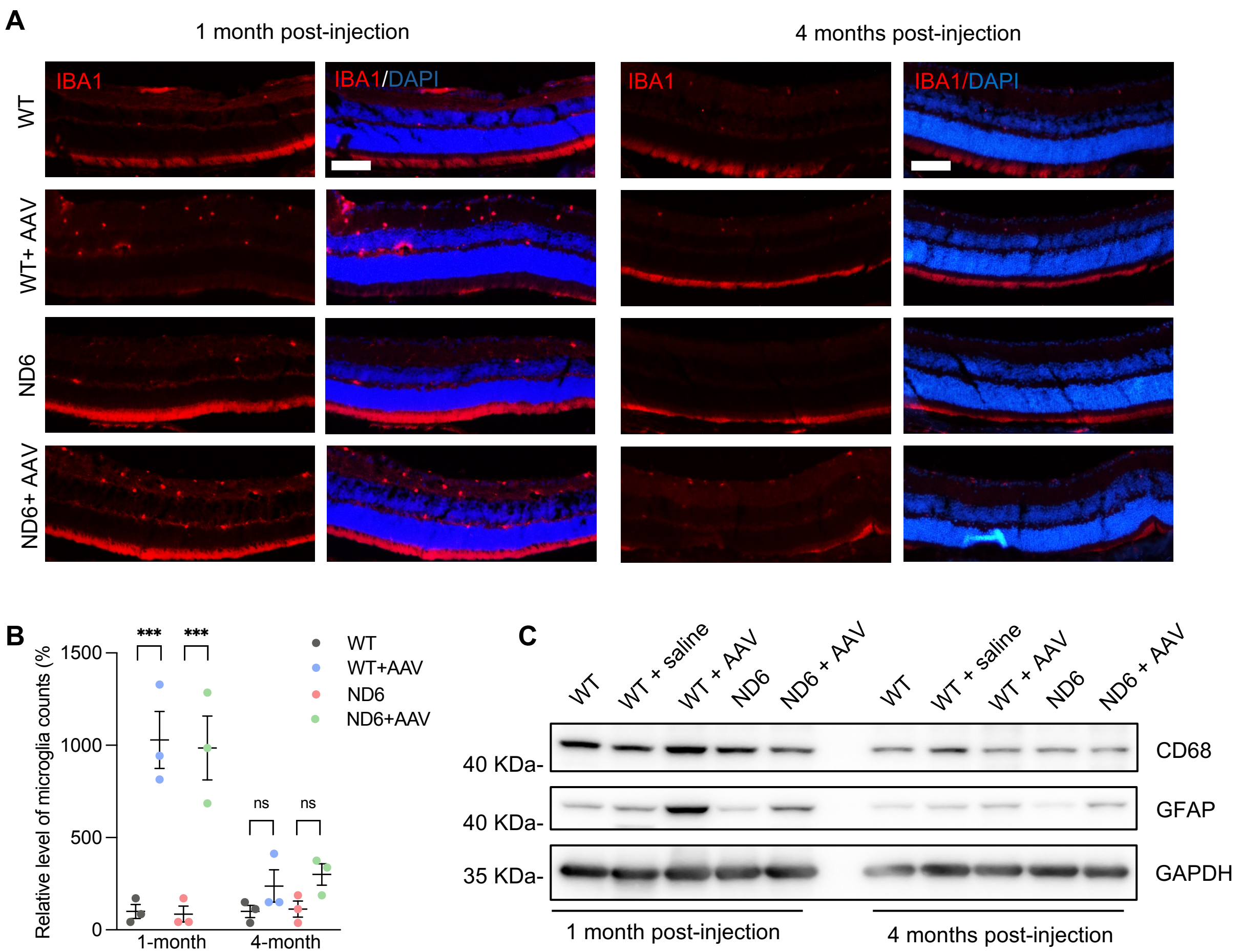

Supplemental Figure 6 (related to Figure 2). Analysis of retinal inflammation. (A) Immunofluorescence staining of cryo-section showing retinal section at 1 and 4 months post-injection stained with IBA1 (red) with DAPI (blue). Scale bar: 100 µm. **(B)** Quantification of the number of microglia in various mice (n = 3 mice). **(C)** Western blot analysis of total retinal lysates from mice retinas at 1 month and 6 months post-injection. Proteins were separated by SDS-PAGE and immunoblotted with antibodies against inflammation markers (GFAP, CD68), the transgene tag (FLAG). GAPDH was used as a loading control. Data are shown as mean ± SEM. ***P < 0.001 by one-way ANOVA followed by Bonferroni post hoc test. ns, no significant.

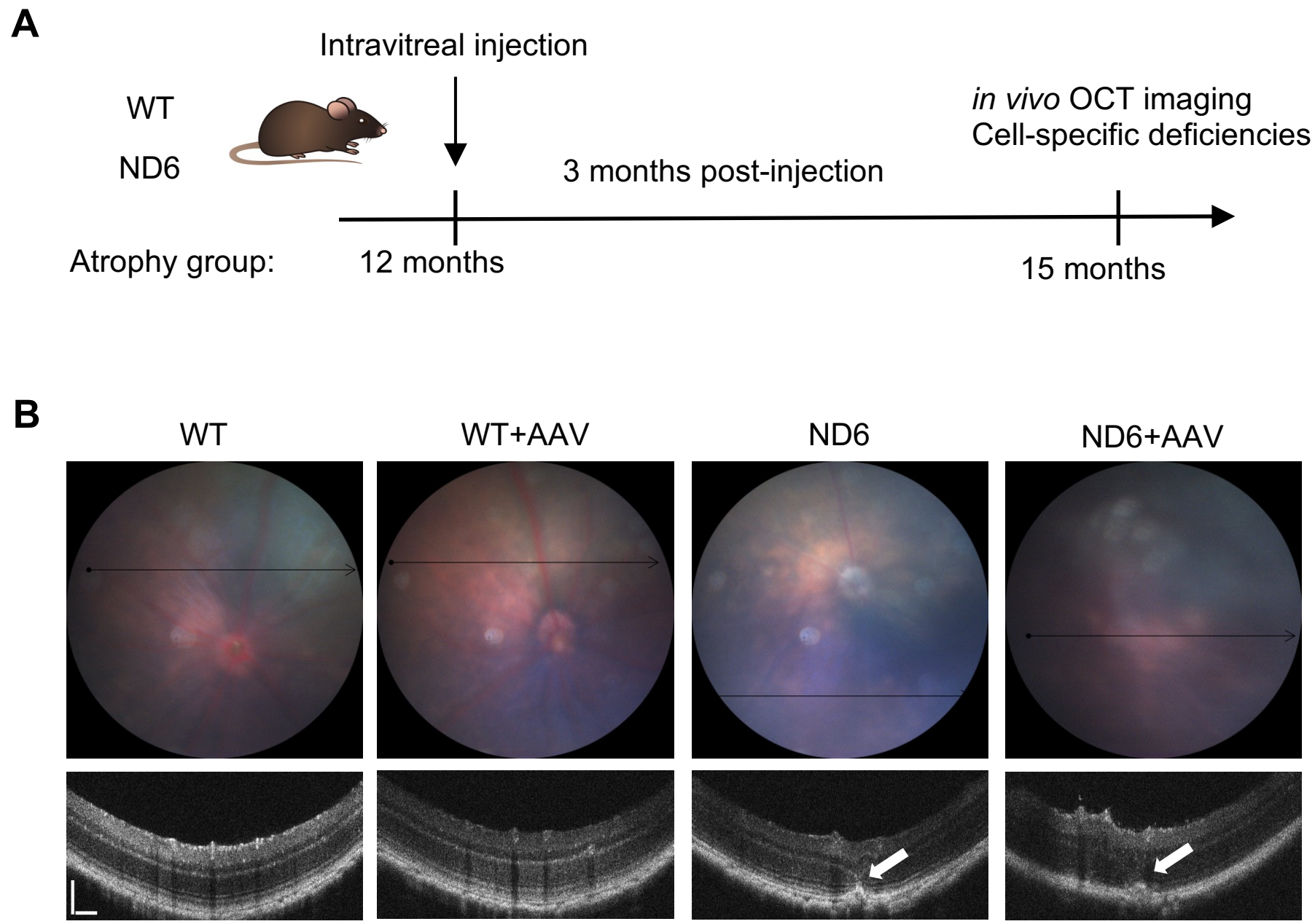

Supplemental Figure 7 (related to Figure 2). Therapeutic evaluation of AAV2-ND6 in 12-month-old mice. (A) Schematic diagram of experimental procedures for the AAV-mediated allotopic ND6 intervention and pathological evaluation in WT and $ND6^{P25L}$ retinas at the atrophy stage. Twelve-month-old WT and $ND6^{P25L}$ mice were intravitreally injected with a high dose of AAV2-ND6. Retinal phenotypes were subsequently assessed at 15 months of age, 3 months post-intervention. **(B)** Representative OCT images of mice retina at 15 months of age. The white arrow indicated abnormalities in the photoreceptor and RPE. Scale bar: 100 μm.

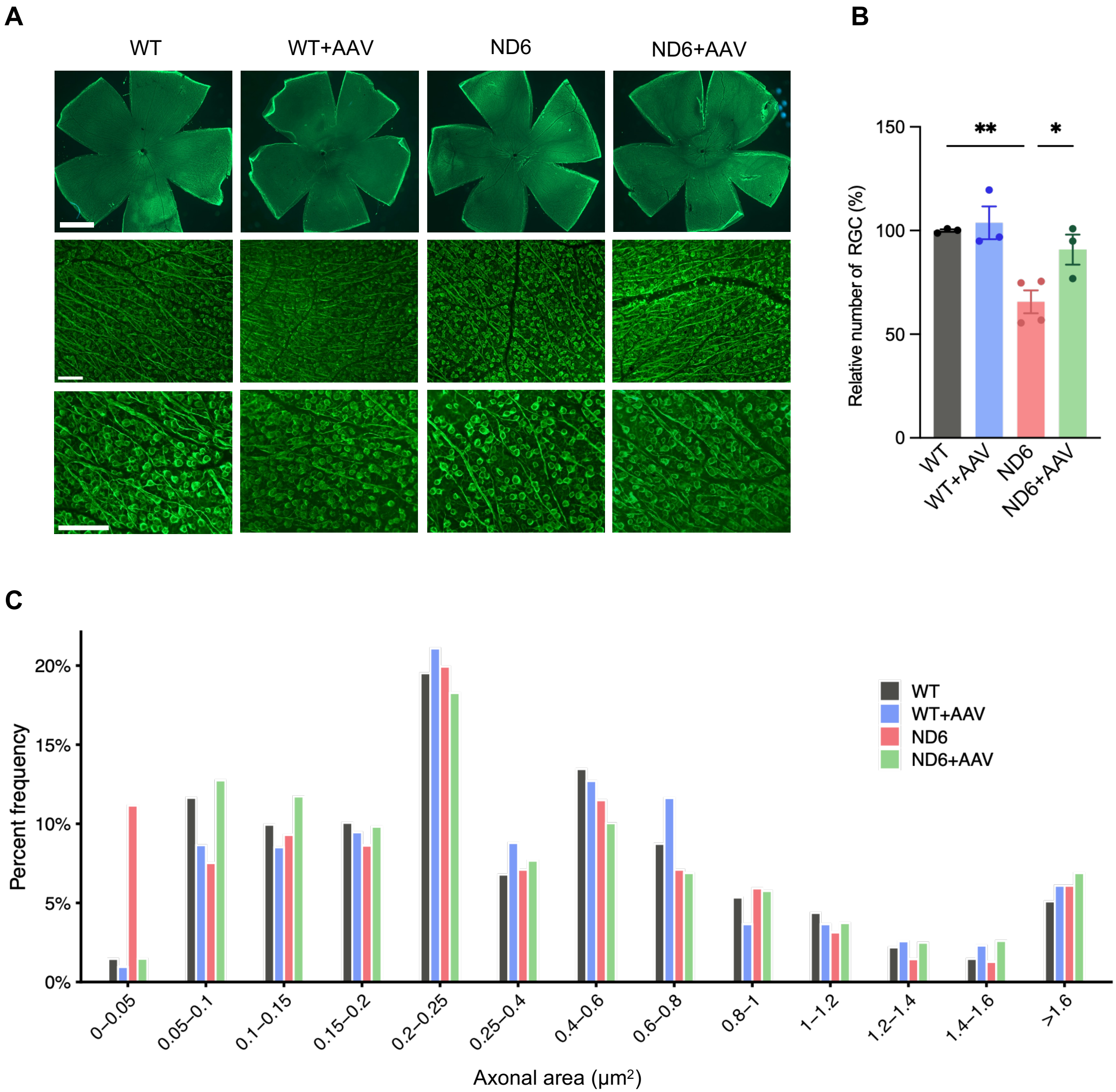

Supplemental Figure 8 (related to Figure 3): Therapeutic evaluation of allotopic ND6 expression in mice retina and optic nerve. (A) Whole-mount retina stained with anti-β-tubulin III antibody. scale bar: 1 mm (upper panel); 100 μm (middle and lower panel). **(B)** The number of RGCs measured from β-tubulin III-positive cells in various mice (n = 3–4 mice). Data are shown as mean ± SEM. *P < 0.05, **P < 0.01 by one-way ANOVA followed by Bonferroni post hoc test. **(C)** Histograms of percentage distribution of optic nerve axon areas in WT, WT+AAV, ND6 and ND6+AAV mice. A total of 825 (WT), 740 (WT+AAV), 1184 (ND6), 887 (ND6+AAV) individual axons were counted.

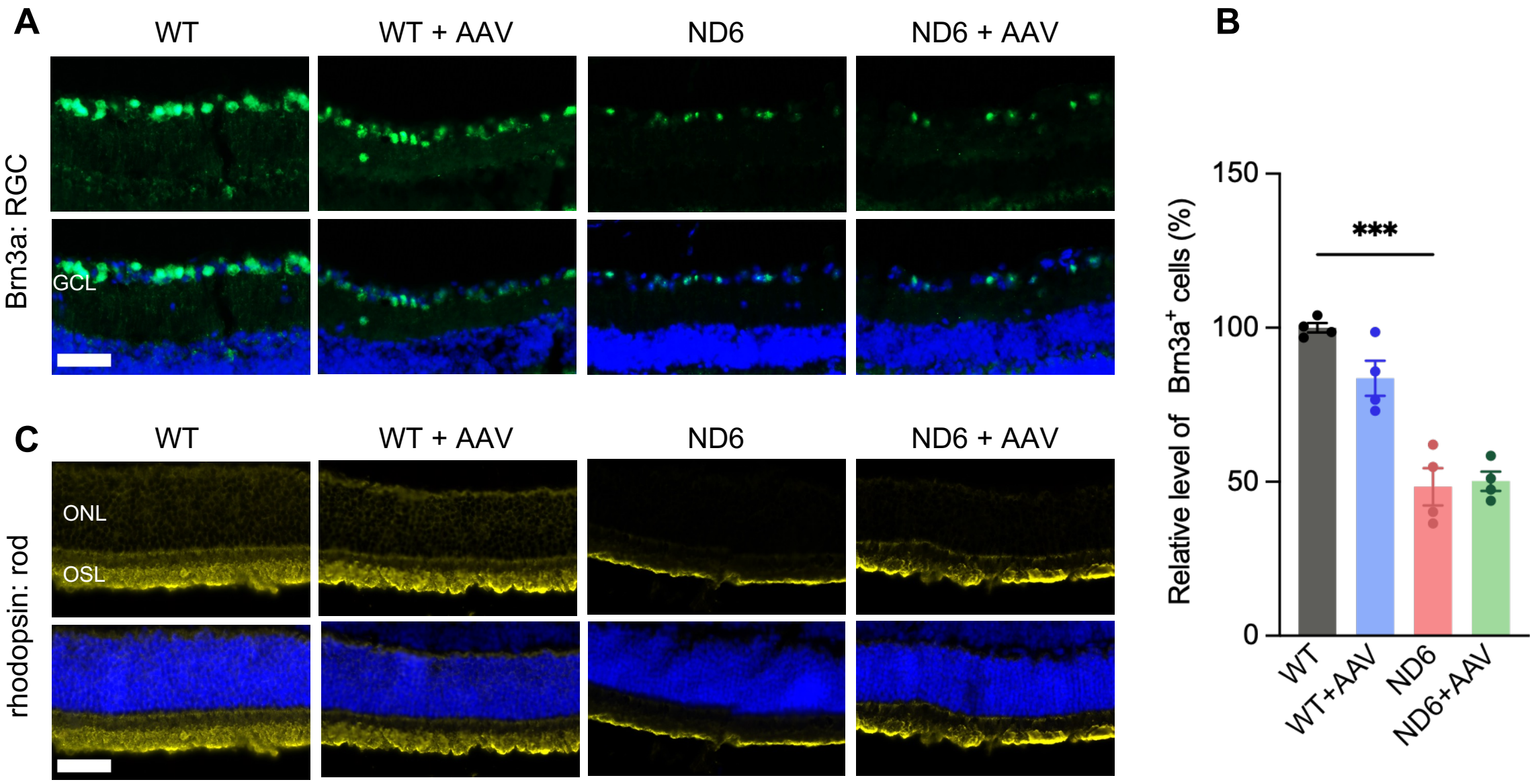

Supplemental Figure 9 (related to Figure 3). Therapeutic evaluation of AAV2-ND6 in mutant retina at the atrophy stage. (A) Immunofluorescence staining of cryo-section showing retinal section stained with Brn3a (green) for RGC and DAPI (blue). Scale bar: 50 μm. **(B)** Quantification of Brn3a positive RGC levels (n = 4 mice). Data are shown as mean ± SEM. **(C)** Immunofluorescence staining of cryo-section showing retinal section stained with Rhodopsin (yellow) and DAPI (blue). Scale bar: 50 μm. ***P < 0.001 by one-way ANOVA followed by Bonferroni post hoc test.

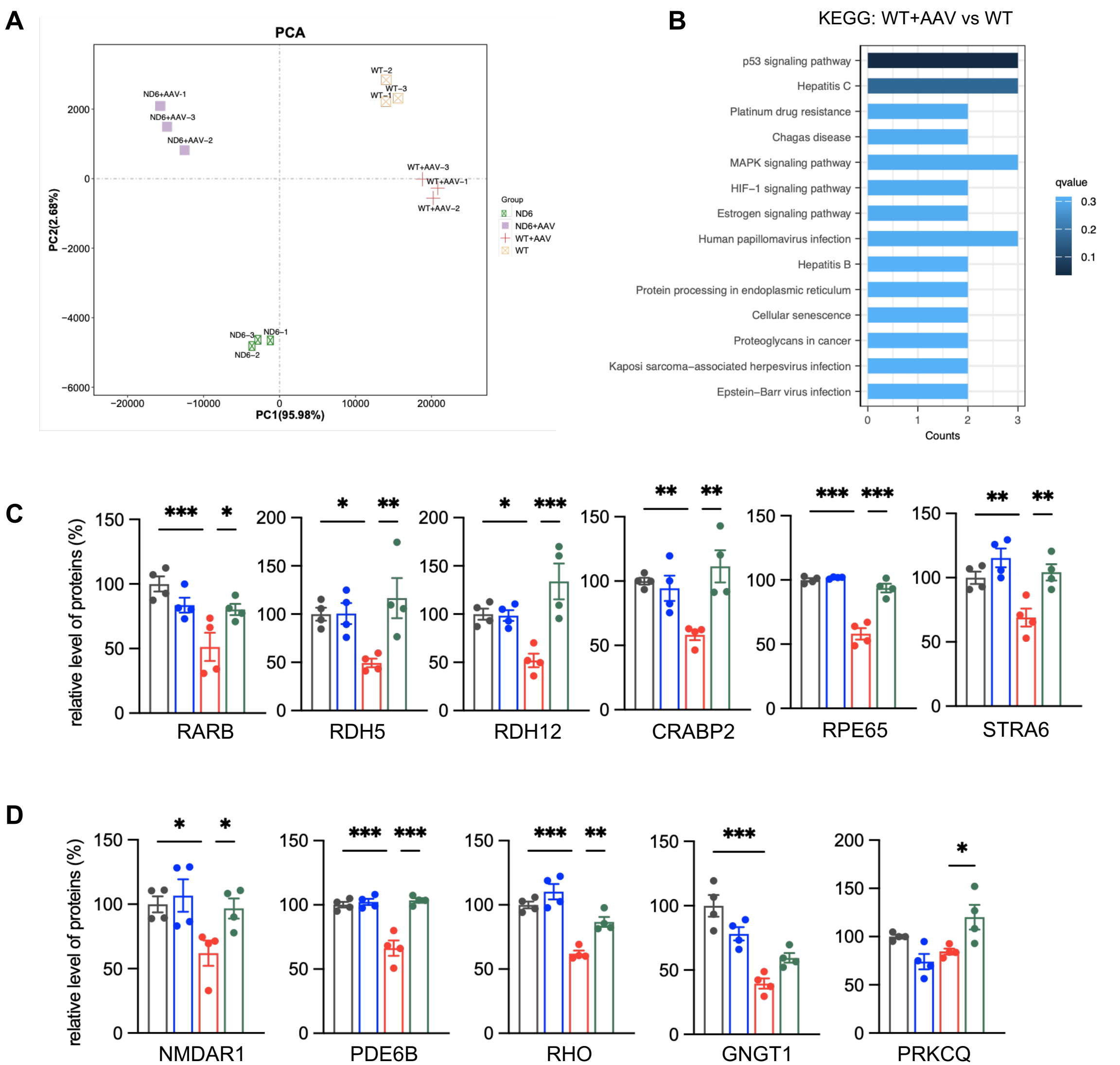

Supplemental Figure 10 (related to Figure 5): Transcriptional changes in mice retina. (A) Principal component analysis (PCA) of mice retinas of different groups. **(B)** Bar plots showing KEGG pathway enrichment for dysregulated genes in WT+AAV mice, as comparing to WT mice. The x axis represented the number of differentially expressed genes and the color indicated q value. **(C)** Quantification of proteins involved in retinol metabolism (RARB, RDH5, RDH12, CRABP2, RPE65 and STRA6) in different mice groups. Representative of 4 independent experiments. Data are as shown as mean ± SEM. **(D)** Quantification of proteins involved in retinol metabolism (NMDAR1, PDE6B, RHO, GNGT1, PRKCQ) in different mice groups. Representative of 4 independent experiments. Data are as shown as mean ± SEM. **P* < 0.05; ***P* < 0.01; ****P* < 0.001 by one-way ANOVA followed by Bonferroni post hoc test. PC, Principal component; KEGG, Kyoto Encyclopedia of Genes and Genomes.

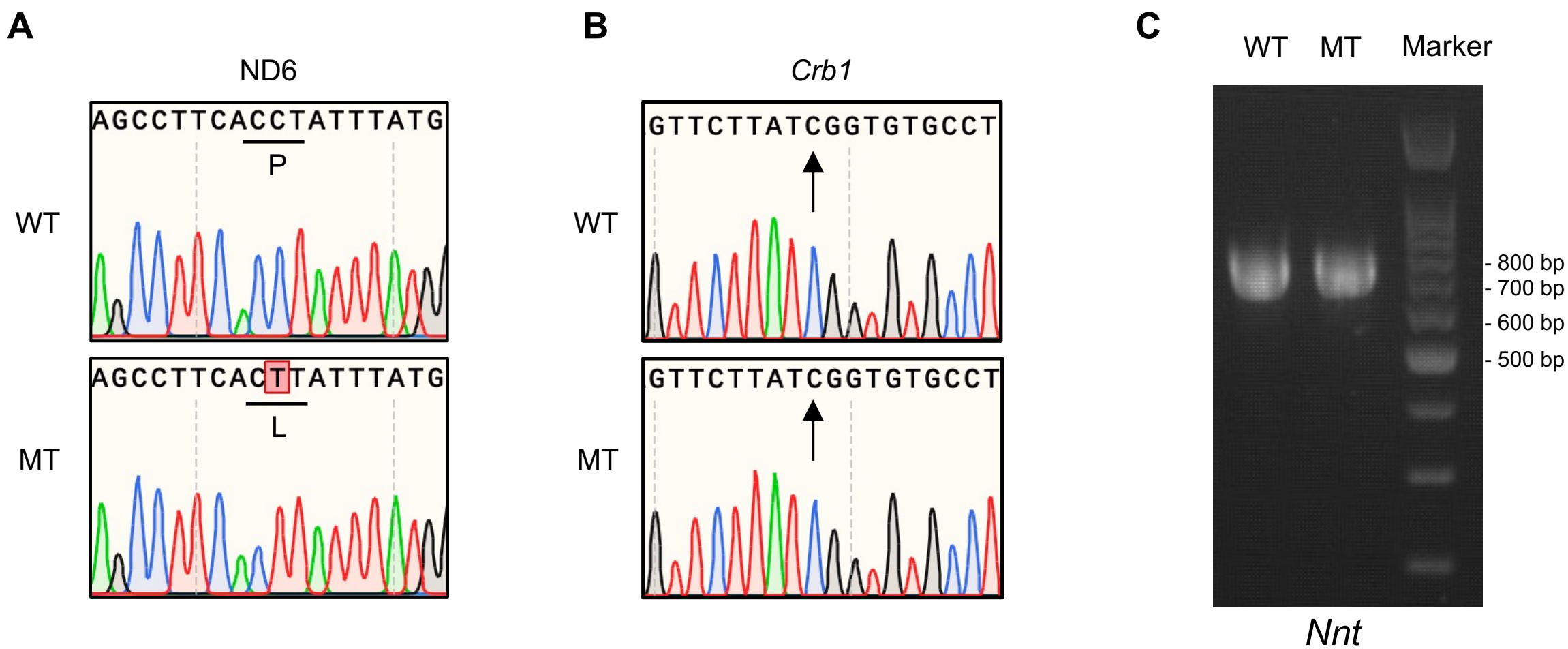

Supplemental Figure 11: Genotyping of ND6^{P25L} mice. (A) Representative Sanger sequencing chromatograms confirming the ND6^{P25L} mutation in mutant mice. **(B)** Representative Sanger sequencing chromatograms of the *Crb1* locus in WT and ND6^{P25L} mice, showing the absence of the rd8 mutation. **(C)** Representative PCR analysis of the *Nnt* locus in WT and ND6^{P25L} mice. The mutant *Nnt* allele characteristic of the C57BL/6J background produced a 743 bp amplicon, whereas a potential wild-type *Nnt* allele derived from C57BL/6N introgression would produce an ~570 bp amplicon. WT: wild-type; MT: ND6^{P25L} mutant.

Table S2: **Oligonucleotides for mouse genetic analysis**

Primer name	Sequence (5'-3')	Description
**Mouse genotyping**		
ND6-F:	CCCCCTAAATAAATTAAAAAA	Product length: 825 bp
ND6-R:	ATAATAAATGGTAAGATGAAG	
Crb1-F	GGTGACCAATCTGTTGACAATCC	Product length: 434 bp
Crb1-R	GGTGACCAATCTGTTGACAATCC	
Nnt-WT-F	GGGCATAGGAAGCAAATACCAAGTTG	Product length:
Nnt-MT-F	GTGGAATTCCGCTGAGAGAACTCTT	Mutant allele: 743 bp;
Nnt-R	GTAGGGCCAACTGTTTCTGCATGA	Wild-type allele: ~570 bp
**qPCR**		
hND6-F	GCAAACCCTCCCCAATTTACG	Product length: 102 bp
hND6-R	CCATCAGCCCCATGTAGCC	
β-actin-F	GGCTGTATTCCCCTCCATCG	Product length: 154 bp
β-actin-R	GGCTGTATTCCCCTCCATCG	
Rpp30-F	TCCAGTGTGCAAGAAAGCTAAATG	Product length: 62 bp
Rpp30-R	GGCAGTGCGTGGAGACTCA	

Table S3. The information of the applied primary and secondary antibodies

Antibodies	SOURCE	IDENTIFIER	Application
Mouse anti-GAPDH	ABclonal	Cat#: AC033	WB
Rabbit anti-FLAG	ABclonal	Cat#: AE092	WB, IHC-Fr
Mouse anti-FLAG	ABclonal	Cat#: AE005	WB, IHC-Fr
Rabbit anti-CD68	ABclonal	Cat#: A24386PM	WB
Rabbit anti-GFAP	ABclonal	Cat#: A19058	WB
Rabbit anti-RARB	ABclonal	Cat#: A4535	WB
Rabbit anti-RDH5	Abcam	Cat#: ab200197	WB
Rabbit anti-RDH12	Proteintech	Cat#: 13289-3-AP	WB
Rabbit anti-CRABP2	ABclonal	Cat#: A6119	WB
Rabbit anti-STRA6	Proteintech	Cat#: 22001-1-AP	WB
Rabbit anti-RPE65	ABclonal	Cat#: A9615	WB
Rabbit anti-PDE6B	ABclonal	Cat#: A6942	WB
Rabbit anti-GNGT1	ABclonal	Cat#: A15675	WB
Rabbit anti-NMDAR1	ABclonal	Cat#: A7677	WB
Rabbit anti-RHO	ABclonal	Cat#: A7245	WB
Rabbit anti-PRKCQ	ABclonal	Cat#: A5834	WB
Rabbit anti-ND1	Abcam	Cat#: AB181848	WB
Rabbit anti-ND6	ABclonal	Cat#: A17991	WB
Rabbit anti-NDUFS2	ABclonal	Cat#: A12858	WB
Rabbit anti-NDUFA13	ABclonal	Cat#: A18071	WB
Rabbit anti-SOD1	Proteintech	Cat#: 10269-1-AP	WB
Rabbit anti-SOD2	ABclonal	Cat#: A19576	WB
Rabbit anti-Catalase	Proteintech	Cat#: 21260-1-AP	WB
Rabbit anti-CYTC	Proteintech	Cat#: 66264-1-Ig	WB
Rabbit anti-BAX	ABclonal	Cat#: A19684	WB
Rabbit anti-BAD	ABclonal	Cat#: A24658	WB
Rabbit anti-BCL-2	ABclonal	Cat#: A19693	WB
Rabbit anti-CASP3	Proteintech	Cat#: 19677-1-AP	WB
Rabbit anti-CASP9	Proteintech	Cat#: 66169-1-Ig	WB
Rabbit Anti-SDHA	Proteintech	Cat#: 14865-1-AP	WB
Rabbit Anti-SDHC	ABclonal	Cat#: A22280	WB
Rabbit Anti-TOM20	ABclonal	Cat#: A19403	WB
Anti-Mouse IgG	Beyotime	Cat#: A0216	WB
Anti-Rabbit IgG	Beyotime	Cat#: A0208	WB
Rabbit anti-Brn3a	Abcam	Cat#: ab245230	IHC-Fr
Rabbit anti-Vimentin	ABclonal	Cat#: A19607	IHC-Fr
Mouse anti-Rhodopsin	Abcam	Cat#: ab98887	IHC-Fr
Rabbit anti-TUBB3	ABclonal	Cat#: A1791	IHC-Fr
YSFluor™ 488 Anti-Rabbit	YESAON	Cat#: 33106ES60	IHC-Fr
YSFluor™ 594 Anti-Mouse	YESAON	Cat#: 34112ES60	IHC-Fr
