# Supplementary material for: Allotopic expression of ND6 restores vision in a mitochondrial disease model
Source: JCI Insight. 2026 Sep 8;11(19):e209108. doi: 10.1172/jci.insight.209108 (PMC13726423; doi:10.1172/jci.insight.209108)

Figure 1B

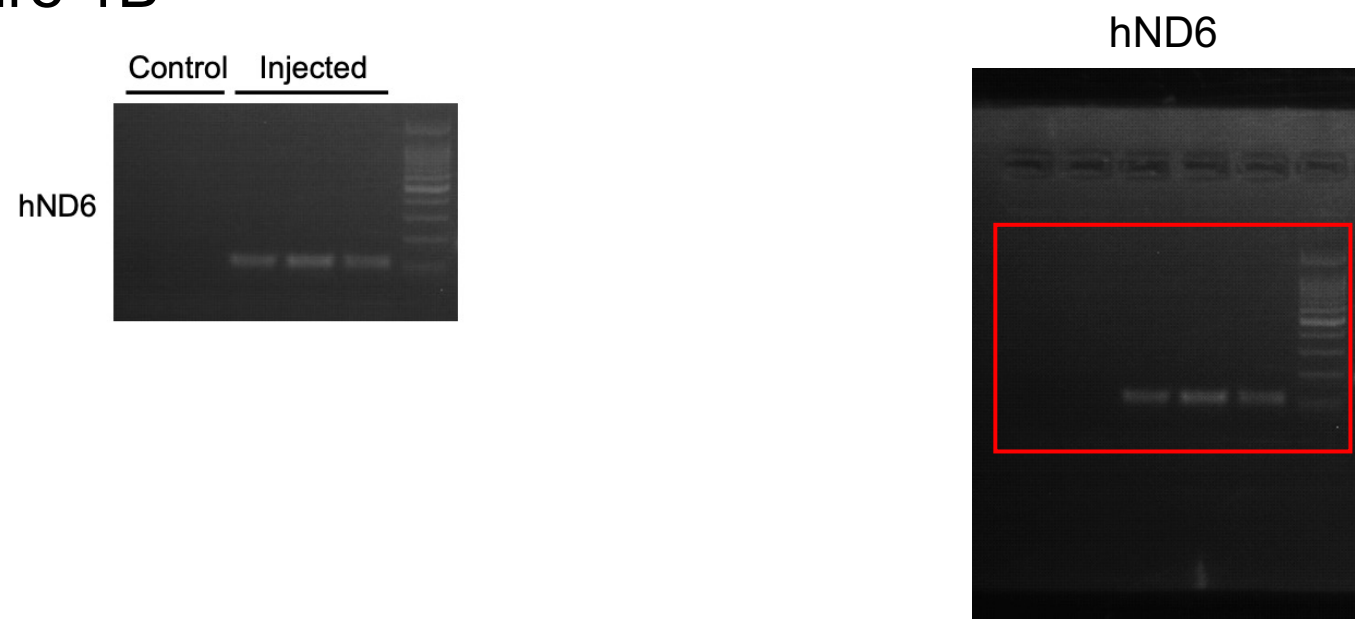

Figure 1D

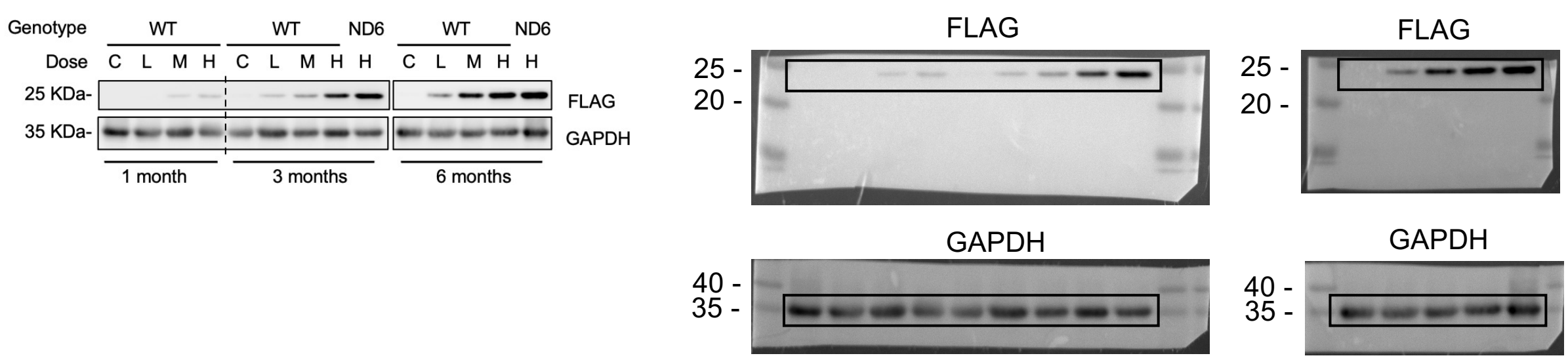

Figure 1F

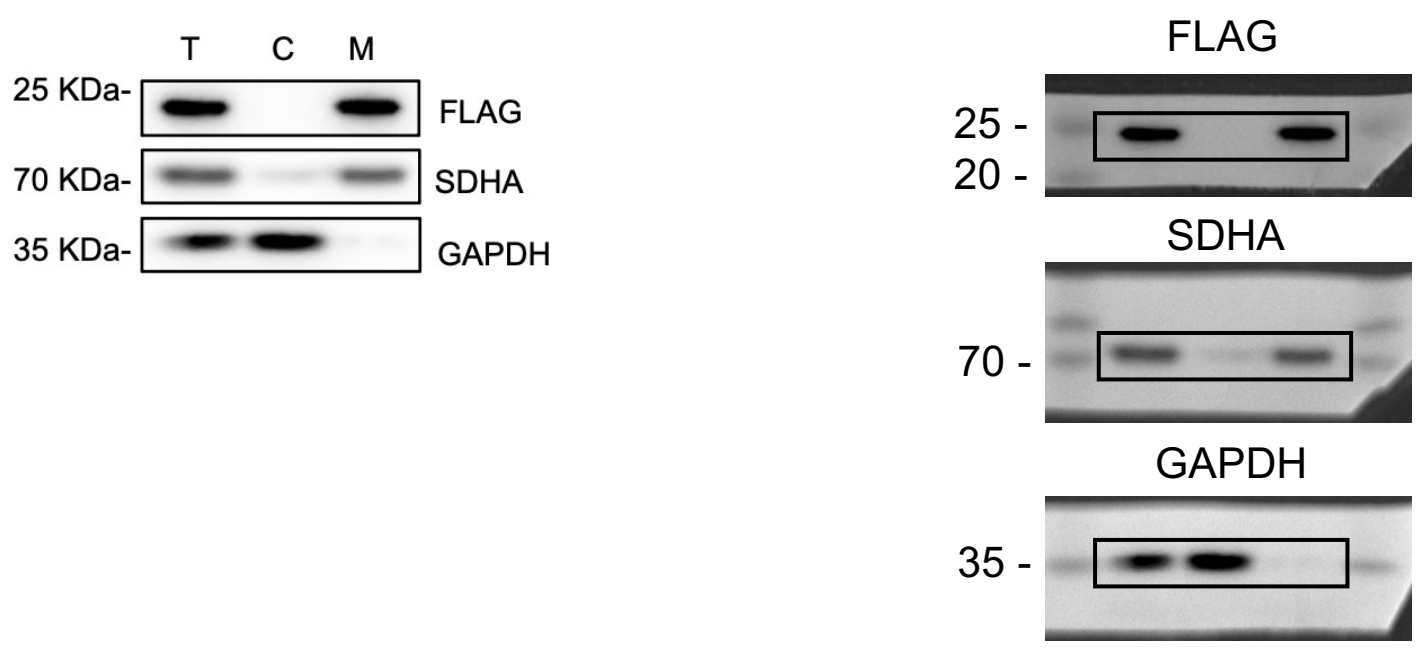

Figure 1G

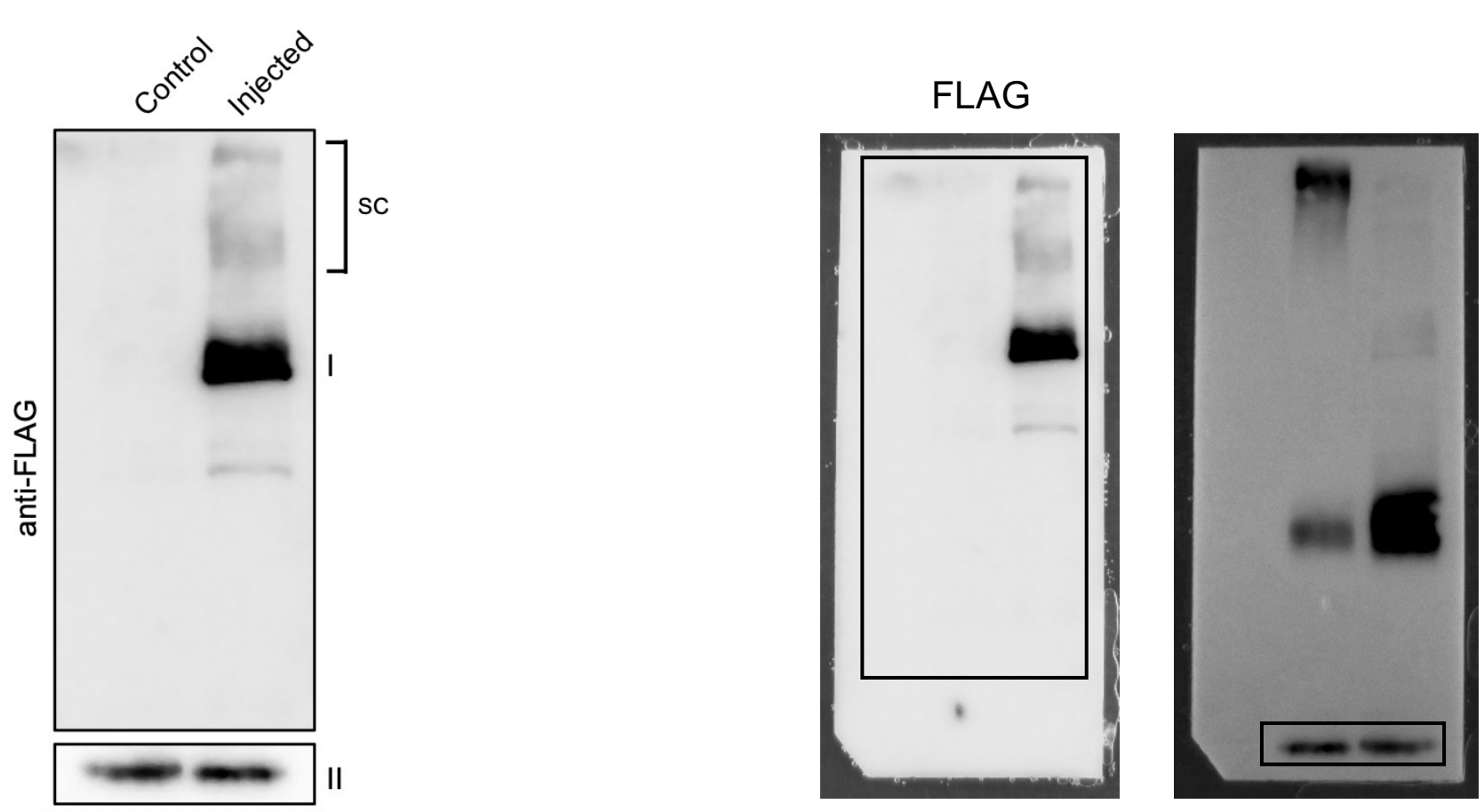

Figure 5F

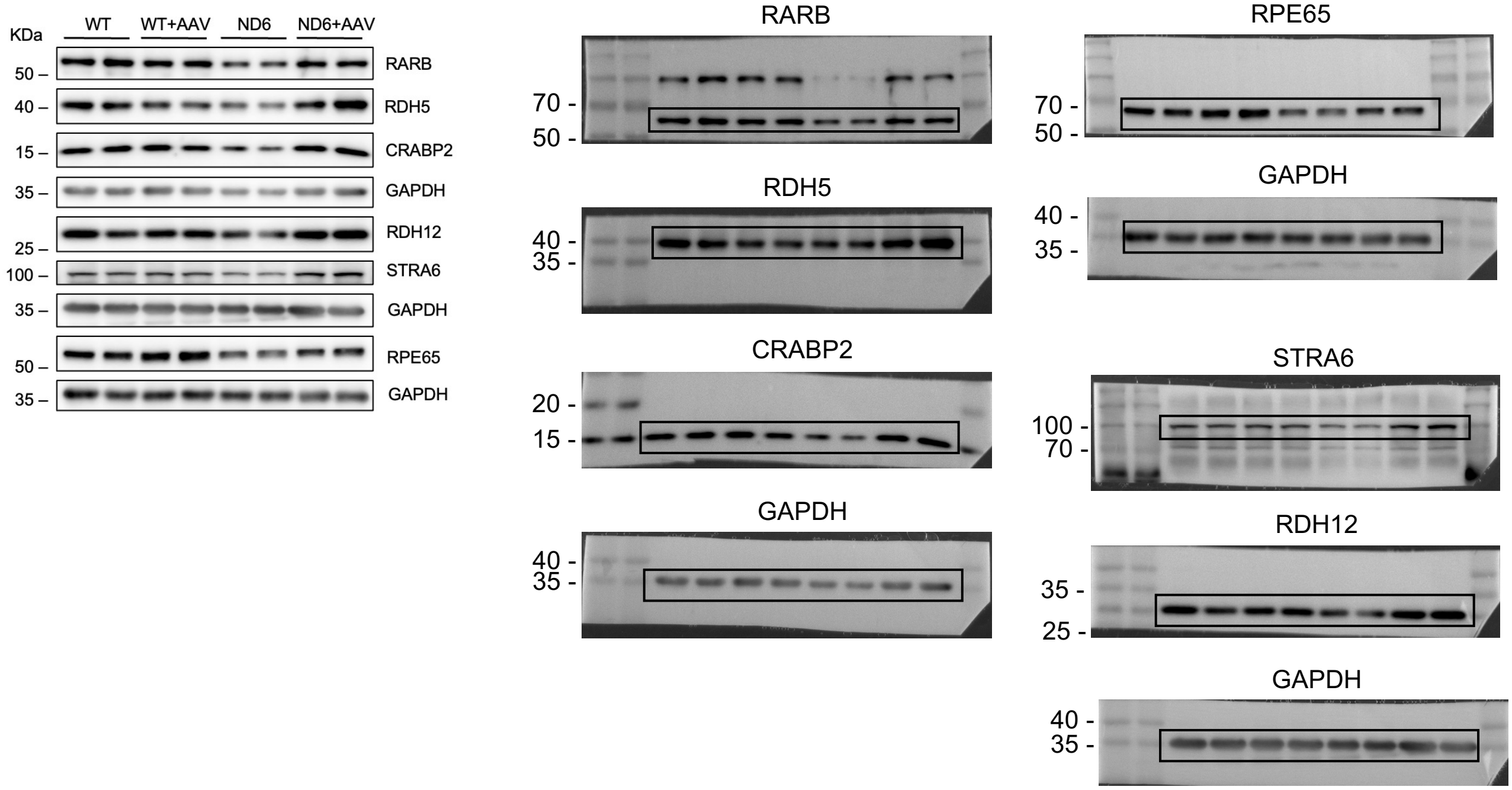

Figure 5G

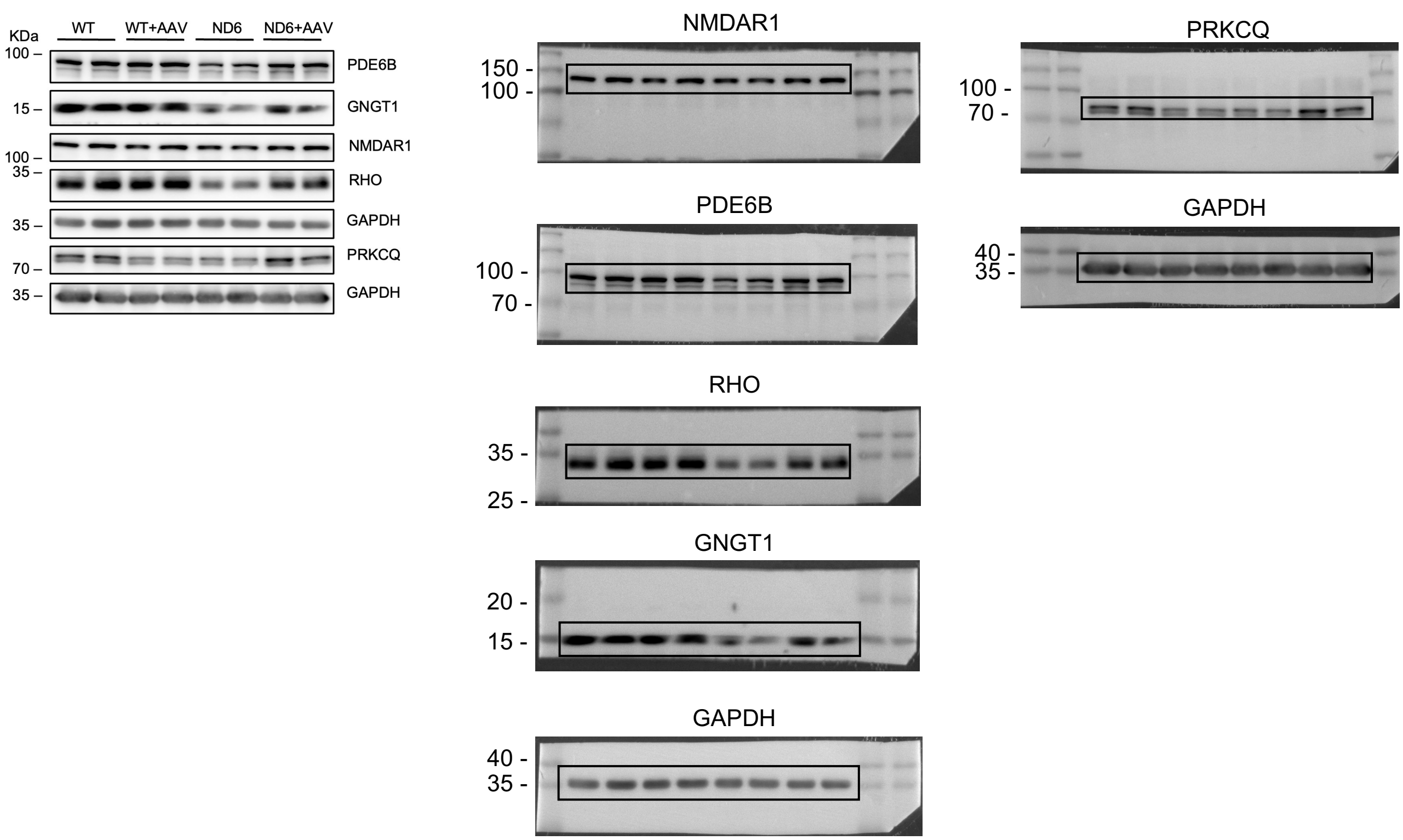

Figure 6A

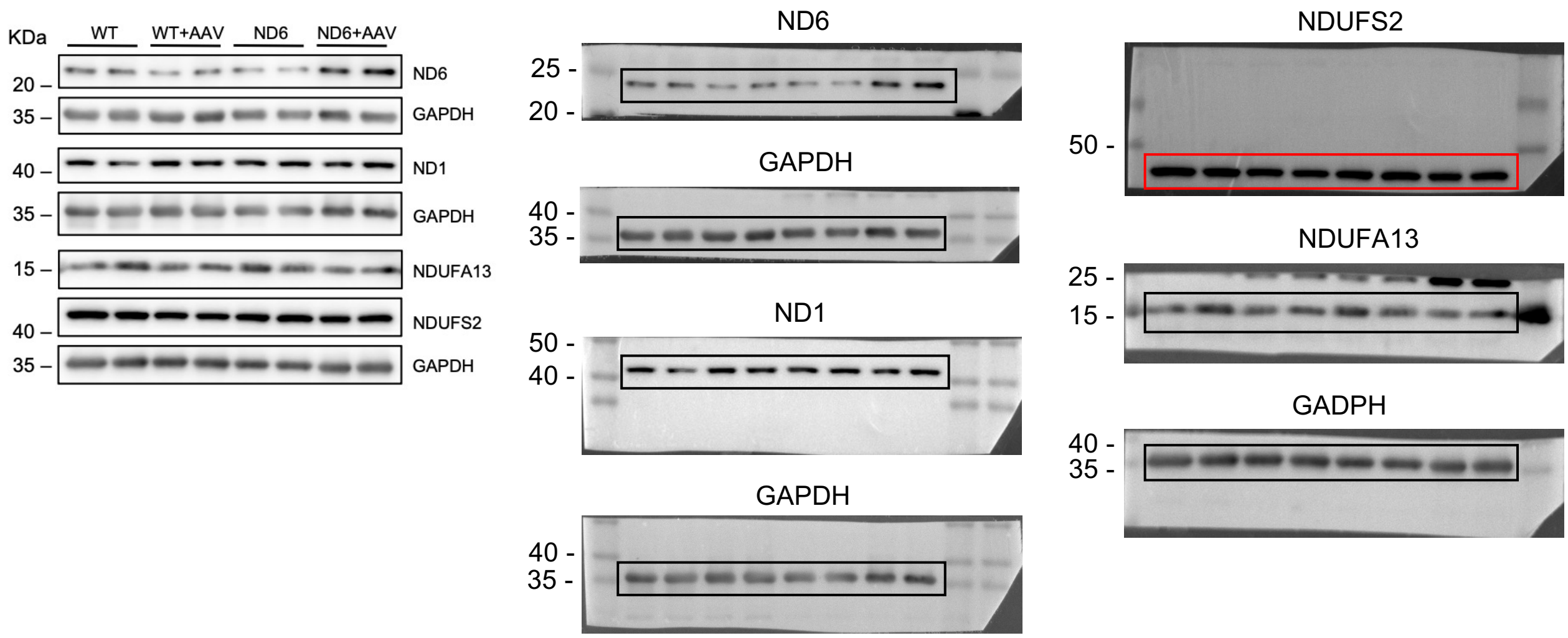

Figure 6G

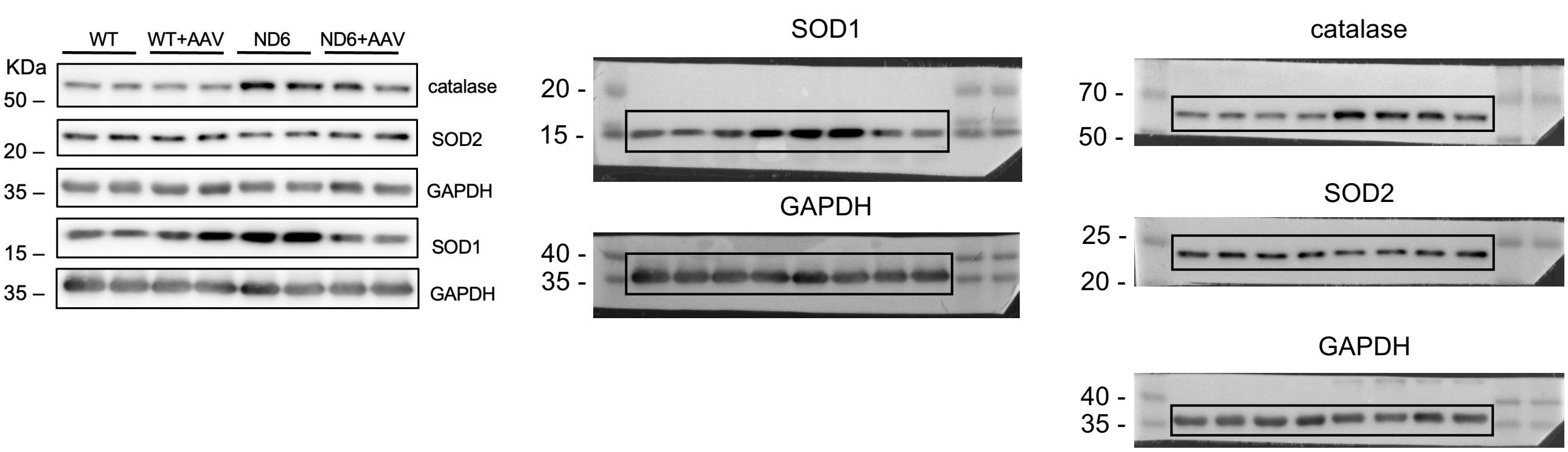

Figure 7D

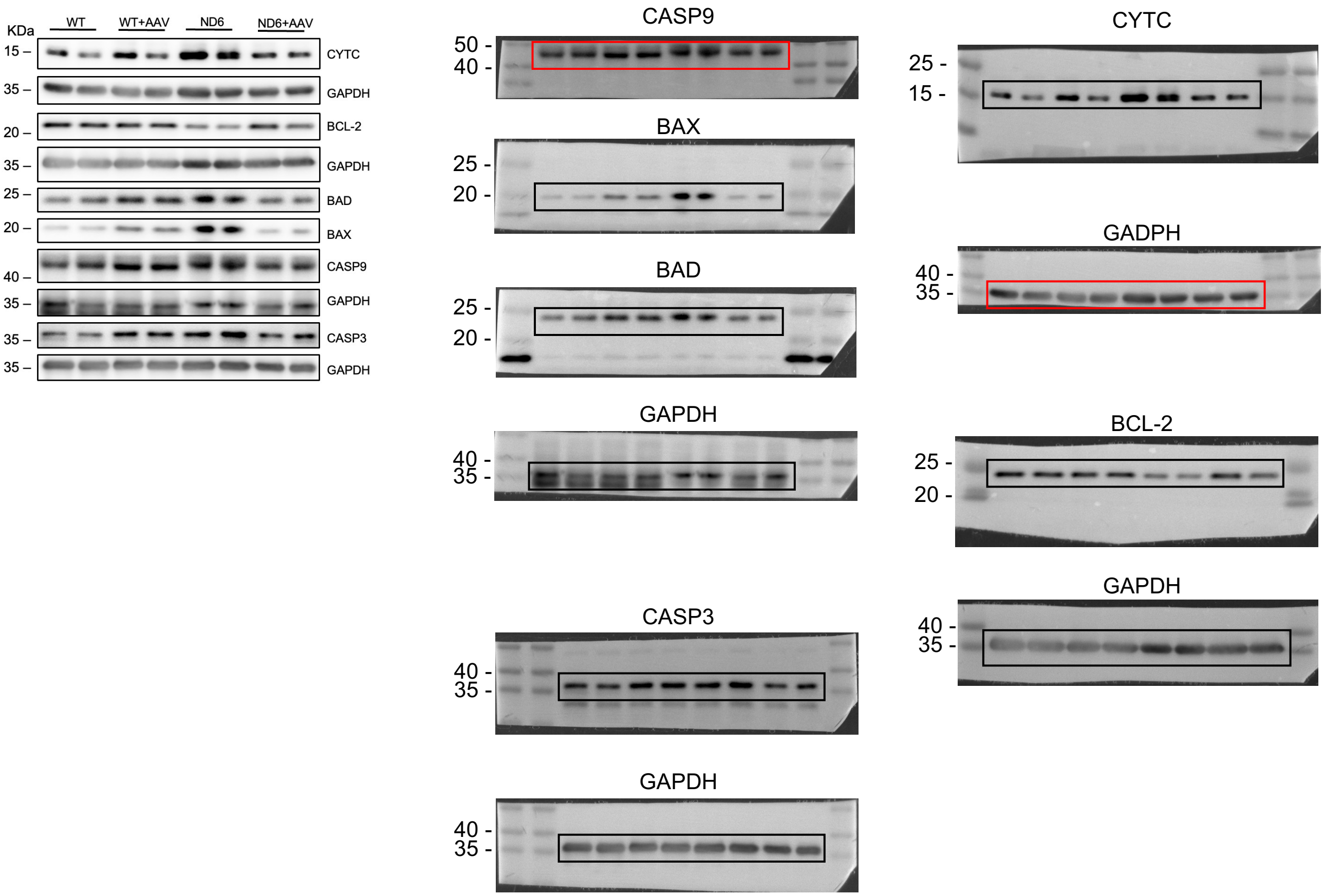

KDa
WT
WT+AAV
ND6
ND6+AAV
15 –
CYTC
35 –
GAPDH
20 –
BCL-2
35 –
GAPDH
25 –
BAD
20 –
BAX
40 –
CASP9
35 –
GAPDH
35 –
CASP3
35 –
GAPDH
CASP9
50 -
40 -
BAX
25 -
20 -
BAD
25 -
20 -
GAPDH
40 -
35 -
CASP3
40 -
35 -
GAPDH
40 -
35 -
CYTC
25 -
15 -
GADPH
40 -
35 -
BCL-2
25 -
20 -
GAPDH
40 -
35 -

Supplemental Figure 1C

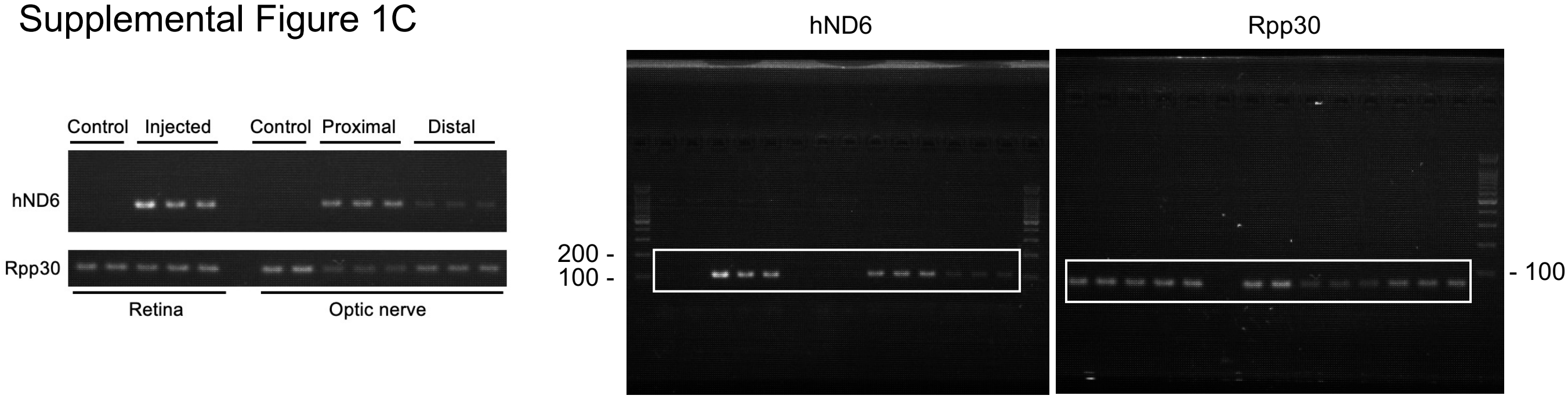

Supplemental Figure 6C

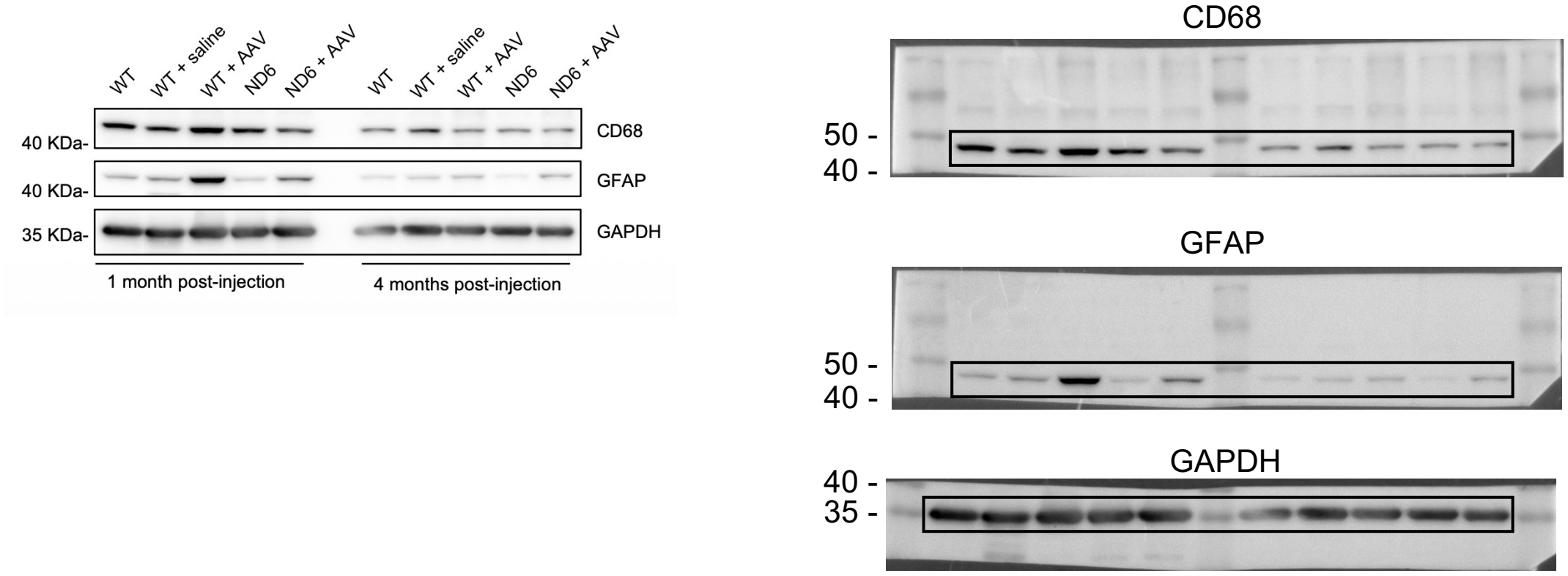

Supplemental Figure 11C

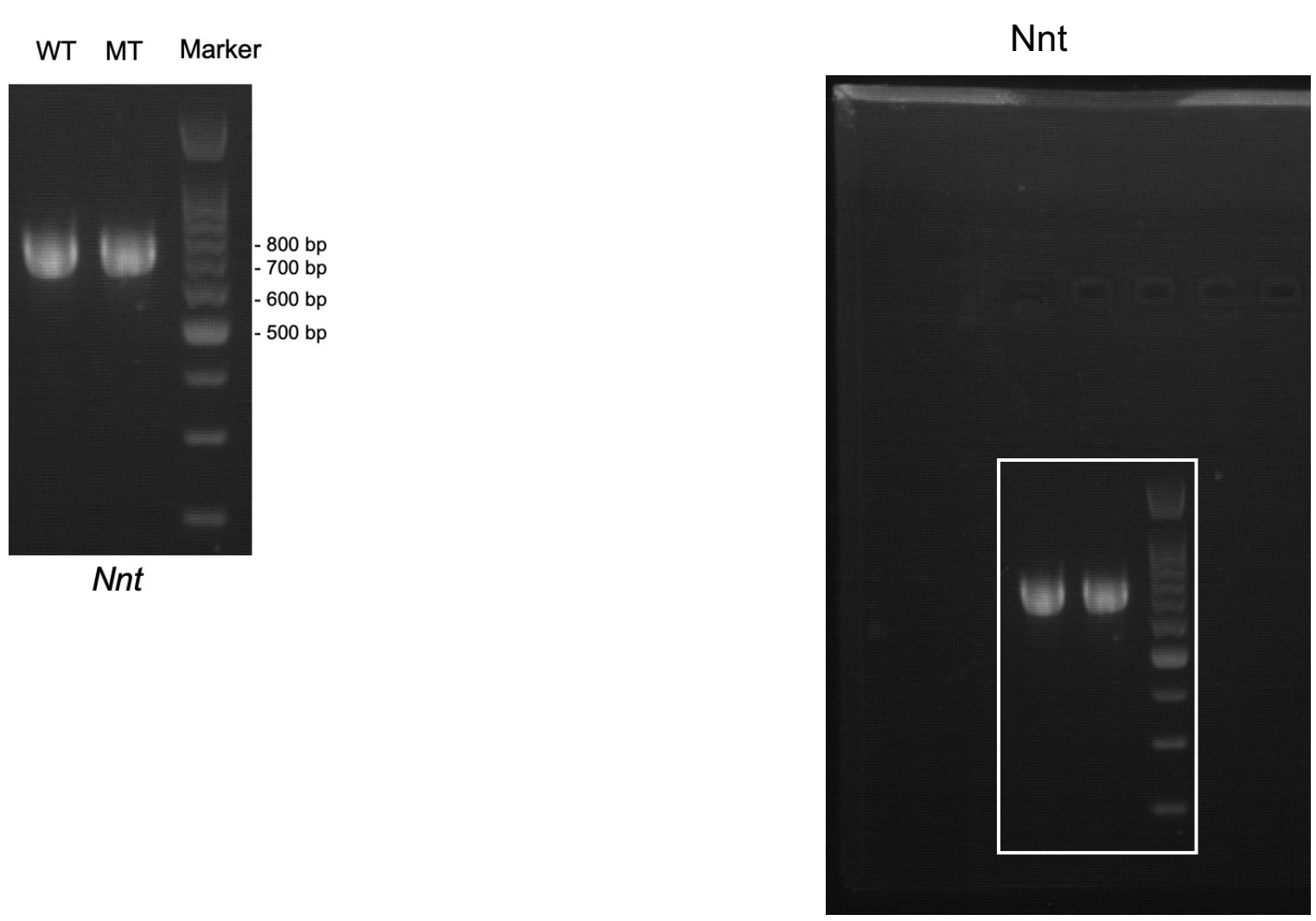

Supplement: Supplementary file 2 [file jciinsight-11-209108-s263.pdf]
